# Six of one, half dozen of the other: Suboptimal prioritizing for equal and unequal alternatives

**DOI:** 10.3758/s13421-022-01356-5

**Published:** 2022-10-12

**Authors:** Warren James, Amelia R. Hunt, Alasdair D. F. Clarke

**Affiliations:** 1 Centre for Health Data Science, University of Aberdeen, Aberdeen, UK; 2 School of Psychology, University of Aberdeen, Aberdeen, UK; 3 Department of Psychology, University of Essex, Colchester, UK

**Keywords:** Decision making, Judgment, Reasoning

## Abstract

It is possible to accomplish multiple goals when available resources are abundant, but when the tasks are difficult and resources are limited, it is better to focus on one task and complete it successfully than to divide your efforts and fail on both. Previous research has shown that people rarely apply this logic when faced with prioritizing dilemmas. The pairs of tasks in previous research had equal utility, which according to some models, can disrupt decision-making. We investigated whether the equivalence of two tasks contributes to suboptimal decisions about how to prioritize them. If so, removing or manipulating the arbitrary nature of the decision between options should facilitate optimal decisions about whether to focus effort on one goal or divide effort over two. Across all three experiments, however, participants did not appropriately adjust their decisions with task difficulty. The only condition in which participants adopted a strategy that approached optimal was when they had voluntarily placed more reward on one task over the other. For the task that was more rewarded, choices were modified more effectively with task difficulty. However, participants were more likely to choose to distribute rewards equally than unequally. The results demonstrate that situations involving choices between options with equal utility are not avoided and are even slightly preferred over unequal options, despite unequal options having larger potential gains and leading to more effective prioritizing strategies.

**Supplementary Information:**

The online version contains supplementary material available at 10.3758/s13421-022-01356-5.

A hungry donkey placed equidistant from two bales of hay will starve to death; at least, this is what happens in the “Buridan’s Ass” paradox. Expected utility theory (EUT) posits that a decision maker, such as the donkey in this paradox, will select the option with the highest expected utility—that is, the product of the option’s value as an outcome and the probability of this outcome (Mongin, 1997; Von Neumann & Morgenstern, 1947). The obvious limit to this strategy comes from estimating and comparing the value and probability of outcomes, a process which can be cognitively demanding, or even intractable (see Bossaerts & Murawski, 2017, for a discussion of this). *Bounded rationality* (Simon, 1990) refers to the idea that people make decisions that are optimal given the limitations of their own memory and attention. A less obvious limitation of EUT is that even for simple choices between a limited set of options, such as choosing between two bales of hay, an equivalence of outcomes risks a decision paralysis like that described in the Buridan’s Ass paradox. The current set of experiments explores the extent to which the presence of equivalent options can disrupt decision-making more globally, causing failures in efficient resource allocation.

## Framing and option equivalence

A decision bias applied to options with the same expected utility can cause one to be selected in spite of their objective equivalence. Contextual factors that can bias decisions are broadly known as framing effects. Several classic examples of framing effects were presented by Kahneman and Tversky (1979) as a challenge to EUT. For example, people tend to make different choices between options when the outcomes are framed in terms of possible gain compared with possible loss. To account for this and several other framing effects, Kahneman and Tversky devised prospect theory as an alternative model to EUT. In prospect theory, options are represented in terms of relative, subjective gains and losses, rather than absolute value, and the psychophysical function describing the relationship between actual and perceived loss or gain is distinct and nonlinear. Although prospect theory provides an appealing alternative to EUT, other modifications to EUT have been proposed that can also account for many framing effects. Regret theory, for example, suggests differences in choice bias for gains and losses can be considered rational once the emotional effect of the choice is factored into its utility (Loomes & Sugden, 1982). That is, included in a given option’s expected utility is not just the loss or gain that is the consequence of the choice, but the emotional value associated with the act of choosing and reflecting on that choice. Neither of these theories, however, specifically address the situation of how to choose between *subjectively* equal alternatives.

Specifically relevant to the Buridan’s Ass dilemma, in contrast, another proposal to account for framing effects is Salience Theory, which adds an element of decision weights to the calculation of utility (Bordalo et al., 2012). In this model, choice variation under different frames reflects the chooser’s attention to different payoffs, some of which might stand out for reasons that are not relevant to their utility. For example, if the donkey spots a particularly juicy-looking bit of hay in one of the two equal piles, and that payoff is given a higher decision weight, this breaks the impasse. In other words, salience could provide an escape from the Buridan’s Ass dilemma, in that two options of equal global objective utility could be valued differently, depending on attention to particular local details. This kind of “local thinking” (e.g., Gennaioli & Shleifer, 2010) could, if the options were unequal, steer the donkey to the less optimal pile of hay, but in the case where they are roughly equivalent, and/or utility is difficult to calculate, salience could bring what could be a lengthy or infinite deliberation to a more efficient end.

In more mechanistic terms, decision-making has been modeled as a dynamic accumulation process, where the expected payoffs of different aspects of options are retrieved and compared sequentially and in a random order, until a threshold is reached (e.g., decision field theory; Busemeyer & Townsend, 1993). A multitude of variations of this kind of model have been proposed, but as noted by Teodorescu and Usher (2013), differences between most of these variations in terms of how well they fit decisions and their timing is negligible. One important distinction these authors do draw, however, is whether there is an independent race between the options or a more direct competition. In competitive models, the accumulation of evidence in favor of one option can affect the accumulation rate for the others, and/or the threshold for one choice is set as a difference, relative to the others (e.g., Ratcliff & Smith, 2004). Independent versus competitive models lead to distinct predictions about deliberation time (i.e., reaction time [RT] in a forced-choice task). For independent models, deliberation time is largely independent of the alternatives, but in competition models, the accumulation rate and/or threshold will change systematically with the context of alternative options, leading to changes in RT. In perceptual decision tasks, Teodorescu and Usher (2013) present evidence that the competitive models provide the better fit with empirical data.

The Buridan’s Ass scenario does not pose a problem for independent race models of decision-making. The presence of equal options would not delay accumulation or change the threshold; equally valuable locations would cross the threshold at different times simply because the rate of accumulation is noisy. In contrast, competitive models tend to predict longer deliberation when the differences between options become less obvious; the existence of these delays, as noted above, has been used as evidence that competitive models are a good description of the decision-making process. While it seems unlikely that delays in choice could become infinite in practical terms, other kinds of measurable disruptions could result from equivalent options, such as the development of “superstitious” behaviour (Skinner, 1948)—that is, particular decisions might become favoured because they are mistakenly attributed to coincidental reward.

## The focus-or-divide decision paradigm

A variant of the Buridan’s Ass decision problem can be illustrated as follows:

Imagine a hungry donkey in a herd of other hungry donkeys, and two empty troughs. The donkey does not know which trough the farmer will deposit the hay into. Once the hay has been dropped off, the donkey will want to reach the trough as quickly as possible before the hay is devoured by the others. Where should the donkey wait for the farmer? The donkey could stand midway between the two troughs, and possibly reach either through fast enough to get at least some hay. If the troughs are far apart, though, standing between them will mean most of the hay will be gone before she gets there. Standing close to one trough will give her a 50% chance of getting lots of hay. To maximize how much hay she gets, therefore, the donkey should stand between the troughs when they are close together, and next to one trough when they are far apart.

We call this a *focus-or-divide* dilemma. It is an example of a resource-allocation problem and is a simplified version of a problem we face routinely in daily life: for example, in deciding which projects to try and accomplish in a given timeframe or deciding where to wait for a person when our rendezvous point was vaguely defined. These problems have in common that we have to choose between dividing our resources over multiple options or focusing on one. The optimal decision depends on evaluating available resources and selecting and focusing exclusively on just one option if we do not have the capability to complete or attend to both.

We can formalize the focus-or-divide dilemma with mathematics rather than donkeys. Let *E* represent the participant’s expected accuracy under some behaviour *ɸ*. In the scenario outlined above, we have:

1 $$E\left(\phi \right)={P}_c(A){P}_s\left(A\left|\phi \right.\right)+{P}_c(B){P}_s\left(B\left|-\phi \right.\right)$$

where A and B are two possible tasks, one of which will be selected with probabilities *P*_*c*_*(A)* = 1 − *P*_*c*_*(B)*. We do not know ahead of time which task will be required, but we can choose whether to prioritize one goal over the other (i.e., *ɸ* = 1 or *ɸ* = −1) or equally prepare for both possibilities (*ɸ* = 0). Once we have set *ɸ*, the goal (A or B) is selected, and our chance of succeeding is given by *P*_*s*_*(A*|*ɸ*) or *P*_*s*_*(B*|*ɸ*). In some versions of this task, *ɸ* = −1, 0 or 1 (Morvan & Maloney, 2012), while in others, intermediate levels of prioritization are allowed (Clarke & Hunt, 2016).

Previous work on this choice paradigm has, to our knowledge, been restricted to the case where A and B are equal and symmetric goals: both *A* and *B* are equally likely to be selected (i.e., *P*_*c*_*(A) = P*_*c*_*(B*) = 0.5), while the difficulty of the two tasks, *P*_*s*_(A) = *P*_*s*_(B), has been systematically varied with respect to a parameter Δ:

2 $$E\left(\phi, \Delta \right)={P}_c(A)\;\ {P}_s\left(A\left|\phi, \Delta \right.\right)+{P}_c(B)\;\ {P}_s\left(B\left|-\phi, \Delta \right.\right)$$

**Fig. 1:**
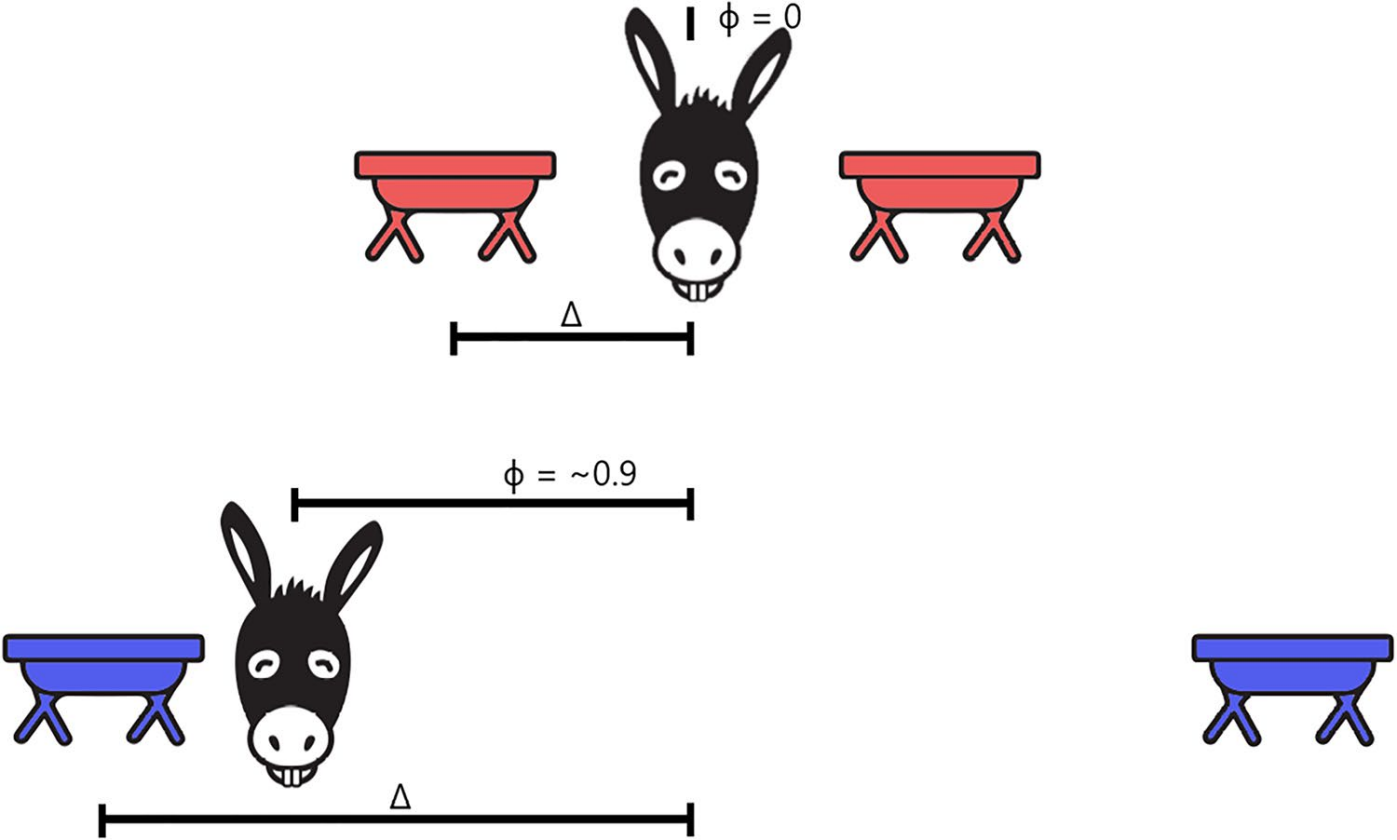
Schematic example of the new donkey dilemma.  Δ measures how difficult the two goals A and B are to complete. In our case, Δ measures the distance from the midpoint to either goal. ɸ represents the position the donkey chooses with respect to the relative probability of success to achieve A or B (from −1 to 1, where 0 is equal). When Δ is small, both A and B are easy to complete, and so the donkey should position itself equally between them, (ɸ = 0)

In our donkey example, Δ represents the distance from the midpoint to each of the two troughs. When Δ is small, such that *P*_*s*_*(A|ɸ* = 0*,* Δ*)* > 0.5, setting *ɸ* = 0 maximizes our expected accuracy^1^. If we increase the difficulty so that *P*_*s*_*(A|ɸ* = 0*,* Δ*)* < 0.5, preparing equally for both potential tasks is no longer optimal, and instead we should opt to gamble on either task A or task B being selected (i.e., *ɸ* = 1 or *ɸ* = −1). Figure 1 shows a schematic representation of the task. In more general terms, the solution to this decision dilemma is to focus on a single goal when the demands of achieving multiple goals exceed the available resources. When both goals are achievable given the constraints, one can focus on achieving both. Throughout this manuscript, we will refer to the success rate that could be expected under the optimal strategy as “optimal accuracy.”

In experiments that present focus-or-divide dilemmas, people consistently demonstrate poor strategies (Clarke & Hunt, 2016; James et al., 2017; James et al., 2019; Morvan & Maloney, 2012). That is, the majority of people, across a broad range of task contexts, do not alter their decisions about whether to pursue one goal or two with the difficulty of achieving the goals. By not following this relatively simple logic, the rate of success can fall far below that which would be expected had the optimal strategy been implemented. The focus-or-divide dilemma is unlikely to push the limits of our cognitive systems. The solution is not difficult to compute, and implementing even an approximation of the optimal solution leads to accuracy that matches optimal accuracy. Participants have been shown to have all the information they need to implement the correct solution (James et al., 2017). Moreover, participants who explicitly understand the optimal solution can implement it easily (Hunt et al., 2019). In other words, this decision problem has an obvious-in-retrospect solution, but something about the way the problem has been presented to participants prevents them from discovering or implementing it.

In the case of the focus-or-divide dilemma we use in this series of experiments, we replicate the methods of Experiment 2 in Clarke and Hunt (2016), in which participants are required to throw a beanbag into one of two hoops. Importantly, they are only told which hoop is the target after they have chosen a standing position. To maximize their chance of successfully hitting one of the hoops, participants should stand between the hoops when they are close together, and next to one hoop when they are far apart. In previous versions of this experiment, participants tend to vary their standing position choices, sometimes standing in the middle, and sometimes closer to one or the other hoop. But the variation in standing position choices did not vary systematically with the distance between hoops (Clarke & Hunt, 2016; James et al., 2017), and as a result, throwing performance fell short of what could have been attained with a more optimal strategy. These are concrete decisions (where to stand) with easily observable outcomes (did the beanbag land in the hoop?). Consequently, we expect the results would generalize more readily to the kinds of decisions people make in daily life, compared with abstract and hypothetical choice problems often used to evaluate human decisions, with participants being expected to imagine they are in a situation where choosing either option would result in one of the two stated outcomes, which may not represent the behaviour of people in real life situations (e.g., Camerer & Hobbs, 2017).

## Prioritization when options are equivalent

In the context of previous experiments using the focus-or-divide dilemma, choosing to focus on one objective entails an arbitrary decision about which objective to focus on. That is, the expected utility is the same regardless of whether participants choose to prioritize *A* or *B*. This decision presents a Buridan’s Ass dilemma that is specific to the “focus” side of the focus-or-divide dilemma. Although participants in previous research do eventually decide upon a course of action, they also do not make rational focus-or-divide choices.

One possible response to an arbitrary choice between two objectives might be to avoid making it altogether, which participants could accomplish by always pursuing both goals, regardless of their difficulty. This behaviour was adopted by a small but consistent subset of participants in Clarke and Hunt’s (2016) experiments. Another possible response could be to overgeneralize—that is, even though only a component of the overall choice problem is arbitrary, participants may make choices that are entirely arbitrary, making their choices variable, but insensitive to the difficulty of the tasks. This describes the behaviour of the majority of participants in the experiments of Clarke and Hunt (2016). In both cases, the presence of a Buridan’s Ass dilemma could be the source of poor decisions in the focus-or-divide dilemma. If so, poor decisions would be limited to conditions involving a choice between two equivalent options. This result would be consistent with competitive models of decision, in which direct competition between equivalent alternatives disrupts global decision-making. Alternatively, the presence of equivalent options may not be disruptive to decision-making in the focus-or-divide dilemma, consistent with independent race models, in which evidence in favour of each of the possible standing positions would accumulate in parallel until one crosses the threshold. Even if the ground truth is that some positions are equally best, noise in the accumulator would push one over the threshold ahead of the others.

Our key question is therefore whether reframing the same decision problem but with nonequivalent options could facilitate decisions. That is, does a Buridan’s Ass dilemma interfere with the process of weighing up alternatives and selecting actions that maximize utility, and can this explain the sub-optimal decisions observed in other studies that involve choices between equal options (e.g., Clarke & Hunt, 2016; James et al., 2017; Morvan & Maloney, 2012)? If so, breaking the symmetry between *A* and *B* will lead participants to make more optimal focus-or-divide decisions. We test this hypothesis in three different ways. In Experiment 1, we introduce a rationale for choosing one of the two options by making one easier than the other. In Experiment 2, we compare the standard condition, in which only one of the potential goals becomes the target, to a condition where both potential goals are known to be the target. Experiment 3 gives participants the opportunity to introduce their own asymmetry to the problem by letting them decide how to split a monetary reward between the two potential targets. Across all three experiments, the basic decision dilemma and the solution remains the same: We present trials where the two targets are close enough that both can be reached from a central position, and trials where they are far enough apart that participants would achieve better accuracy by committing to one or the other. Breaking the symmetry between the two targets has little bearing on the first choice (whether to hedge or commit) but makes a key aspect of second choice (which one to commit to) no longer arbitrary. If this equality between the two goals was the reason for the poor decisions, we should see choices that are closer to optimal when we break the symmetry between goals. If found, this pattern of results would serve two useful functions. First, it would supply an explanation for poor decision-making in previous focus-divide dilemmas, which have until now gone unexplained. Second, it would provide further support for competitive, as opposed to independent, models of decision-making by showing how the disruptive effects of equivalent options can extend into the broader decision-making context.

## Experiment 1: Unequal difficulty

We created an asymmetry between the two potential target locations by making one of the options more difficult—that is, *P*_*s*_*(A|ɸ,* Δ*) ≄ P*_*s*_*(B|ɸ,* Δ*)*. Similar to Clarke and Hunt (2016), we asked participants to decide where to position themselves in order to throw a beanbag into one of two hoops, but one of the hoops was smaller than the other. The participants know the distance between the hoops and their relative size when they decide where to stand, but they do not yet know which of the two hoops is the target. To maximize success, participants should stand closer to the smaller hoop, in proportion to the size difference. Such a strategy would demonstrate that participants are sensitive to the relative size manipulation and can use expected performance to modify their standing position choices. This would be consistent with results from James et al. (2017), who demonstrated that participants have reasonably accurate insight into their own throwing ability in this task. Taking the probability of success for each of the targets into account in choosing a standing position would also be consistent with *spatial averaging* (Chapman et al., 2010), a behaviour observed in visually guided reaching. In these experiments, participants need to begin a reach before a target location is known, and reaching trajectories tend to be spatially weighted to reflect the probability of different locations becoming targets.

If having a reason to choose one hoop over the other facilitates optimal decisions, we should see optimal choices in standing position as the distance between the hoops varies. That is, participants will choose to stand centrally, but slightly closer to the small hoop, when expected accuracy from this central position is greater than 50%. As in Clarke and Hunt (2016), we also included distances where accuracy from a central position would be less than 50%; at these distances, standing next to the smaller hoop will ensure accuracy of at least 50%.

### Methods

#### Participants

There were 21 (four male) participants in Experiment 1. All participants were recruited from the University of Aberdeen community via word of mouth. The protocol for this and all other experiments reported here were reviewed and approved by the Aberdeen Psychology Ethics Committee. None of the participants had taken part in related studies run by our lab.

#### Power analysis

The power analysis was carried out using bootstrapping methods and previously collected datasets. We fit beta distributions to each of the participants in the throwing experiment (2) of Clarke and Hunt (2016) and simulated the hoop size manipulation by shifting the distributions towards the small hoop by 0.05 of the normalized range, where 0 is the center and 1 is the hoop position. We used these distributions to simulate experiments with a range of different sample sizes from 3 to 24. The uncertainty around the estimate of the mean difference between hoop size conditions plateaued around *N* = 15. This demonstrates that the conclusions based on a sample size of at least 15 are highly unlikely to change with any additional participants. Further details of this analysis are presented in the Supplementary Material. The power analysis generalizes to apply to detecting any shift in standing position greater than 0.05 of the normalized range from the center to the hoop—therefore, based on this power analysis, the sample size in this and all subsequent experiments is greater than 15.

#### Equipment

**Fig. 2:**
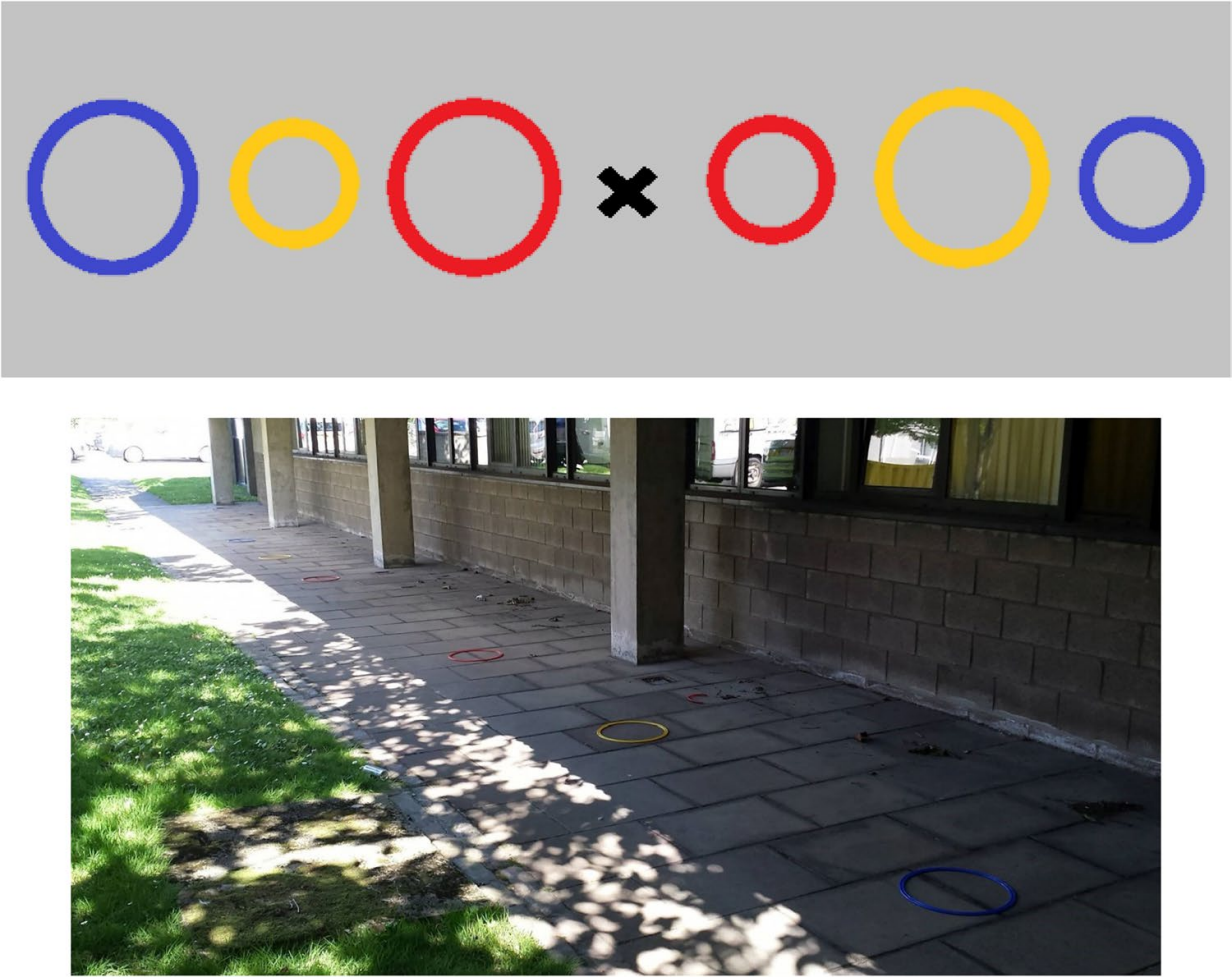
Methods for Experiment 1. The top panel shows a schematic outline of the setup for the second session of Experiment 1. The bottom panel shows the area in which the experiment took place

The experiment was conducted in a sheltered, outside, paved area. Participants were required to throw bean bags into hoops placed on the ground. The paving slabs (each measuring 0.46 cm × 0.61 cm) acted as a convenient unit by which to record the placement of hoops and where participants chose to stand. Figure 2 shows an image of the testing area and setup. The targets were flat plastic hoops of two sizes (diameters of 63.5 cm and 35.5 cm) and three colors (blue, yellow, and red). Beanbag colors matched the hoops (three each of blue, yellow, and red).

#### Procedure

This experiment was based on the throwing task described by Clarke and Hunt (2016) and was conducted over two sessions, conducted on different days at least 1 week apart.^2^ The first session allowed us to measure *P*_*s*_(*A*|*ɸ*) for each participant, while session two involved the focus-or-divide decision paradigm.

##### Session 1 (measuring throwing performance over distance)

The goal of this session was to obtain a throwing performance curve over distance for each participant for the two hoop sizes. The large hoops were tested at a set of seven distances between 7 and 25 slabs (3.22 m–11.5 m). The small hoops were tested at seven distances from 3 to 19 slabs (1.38 m–8.74 m). Participants threw 12 bean bags into each hoop size at each of seven distances, for a total of 168 throws. Two different directions were used (hereby referred to as North and South) with the starting direction counterbalanced across participants. The results of Session 1 (presented in full in the Supplementary Information) were used to model the relationship between *accuracy*, *distance* and *hoop diameter* for each participant using a generalized linear model.

##### Session 2 (choosing a standing position)

Session 2 presented the focus-or-divide dilemma, by asking participants to choose where to stand before the target hoop had been specified. Six hoops, of three different colours, were placed on the paved area (see Fig. 2). The red hoops were always closest together, the yellow hoops further out, and the blue hoops the furthest apart. For each pair of hoops there was a small hoop and a large hoop. The hoop positions in Session 2 were determined by each individual’s throwing ability, to equate overall expected accuracy across participants. To do this, we calculated the distances at which a participant would be 10%, 50%, and 90% accurate for both hoop sizes based on the model of their throwing performance in Session 1. The midpoint of these values was then taken so that there would be a common central point for both sizes of hoops. For example, if a given participant was 50% accurate when the large hoop was 10 slabs away, and 50% accurate when the small hoop was eight slabs away, the small and large hoops would both be placed nine slabs from the centre point in Session 2, to approximate an expected overall accuracy from the centre point of 50%. Each colour pair corresponded to expected throwing accuracy (Red = 90%, Yellow = 50%, Blue = 10%) as measured from an unmarked central position, equidistant from both hoops. Hoop size was alternated.

To sample across a range of separations between hoops, the second block was set up the same way as the first set (with a hoop pair, defined by color, at each of the three separations), but with a different set of separations. The red pair (one large and one small) was positioned one slab closer than their 50% slab (called 50%−1), the yellow at the 50%+1 slabs, and the blue at the 50%+2 slabs. The two sets of three hoop separations were tested in two blocks of 45 trials each, for a total of 90 standing position decisions per participant in Session 2.

On each trial, participants would draw one beanbag at random from a bag. The colour of the beanbag indicated which pair of hoops would be the target for that trial; so, for example, if the participant drew a red beanbag from the bag, they now knew that the two red hoops were the possible targets on that trial. The point of this random draw was to mix up the trial order so that trials with different hoop separations were distributed across the experimental session. The bag contained nine beanbags (three of each colour: red, yellow, and blue). Once thrown, each beanbag was removed from the paved area. After all nine had been removed from the bag and thrown, the bag was refilled. The bag was set off to the side of the paved area so all participants had to return to this location before each trial. Participants were told that they were allowed to stand anywhere they wanted on the paved area. They were also informed that each hoop was equally likely to be the target, and that the order of target hoops had been predetermined in a random fashion. The data recording sheet used by the experimenter included a printed sequence of 90 targets to follow, on which the target on each trial had been independent and randomly selected between north and south hoops. Once participants had stood in their chosen position and informed the experimenter they were ready, their standing position was recorded (in slab units) and they were told which hoop to aim for (as either the “North” or “South” hoop, with the participant reading this off from the predetermined list). The experimenter then recorded throwing accuracy, collected the beanbag, and instructed the participant to draw a new beanbag for the next trial.

#### Analysis

All analyses for this and subsequent experiments were carried out using R (Version 3.4.3; R Core Team, 2016) with the tidyverse collection of packages (Version 1.3.0; Wickham et al., 2019). Our main goal in the current experiment was to assess whether or not standing position decisions improved with unequal hoops sizes, and this question can be addressed through a simple presentation of the data. To keep the results section simple and focused on this question, we present a descriptive analysis of the Session 2 results below. For the purpose of replication or extension of these results, a brief report of the modelling results is presented here with the full reporting of all the data from both sessions in the Supplementary Materials. This analysis made use of the brms package (Version 2.8.0; Burkner, 2017, 2018). Because the descriptive analysis of results addresses the questions the experiments are designed to answer, and the models of the data are complex and lengthy to describe, a similar approach to the results was taken in all the experiments presented in this report.

### Results

#### Standing position

**Fig. 3:**
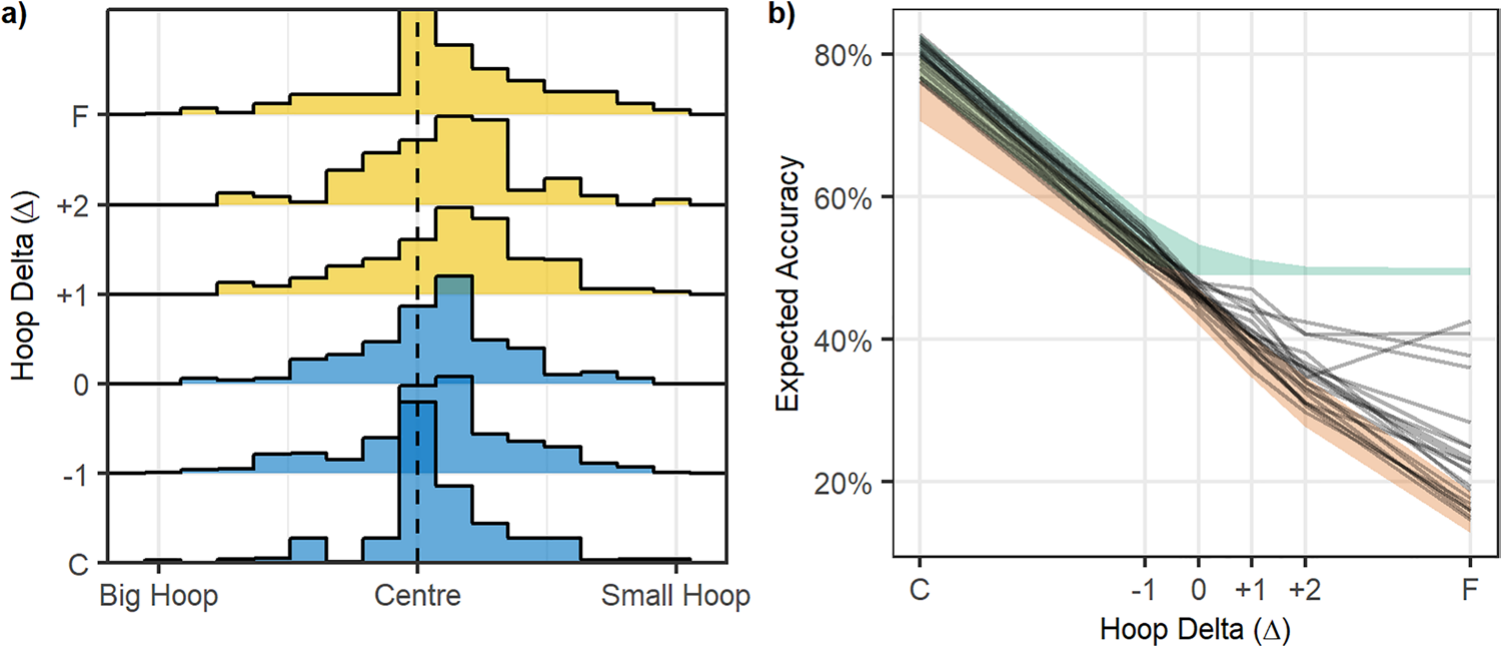
Experiment 1 results. **a** The histograms show the distribution of standing positions for each value of Δ (the distance from the center to each of the hoops). The increments of Δ increase on the *y-*axis from C (the closest distance, where expected accuracy from center is 90%) to F (the farthest distance, with expected accuracy of 10%), with the 0 point being where the expected accuracy from center is 50%. The colour of the histogram indicates the optimal strategy, with blue representing cases when the participants should have stood near the centre, and yellow the cases where standing next to the small hoop was the best strategy. **b** The black lines show expected accuracy for each participant for each of the six hoop distances (with close (C) to far (F) distances now shown on the *x*-axis). The green shaded area shows the range of optimal accuracy for this group of participants, and the red shows their minimum accuracy (the range of accuracy that would be expected if participants chose the least optimal standing position). (Colour figure online)

The results, summarized in Fig. 3a, show the effect of the manipulation of hoop size on standing position choices. Participants tended to stand closer to the smaller, harder-to-hit hoop than the larger one. This demonstrates that participants are motivated and capable of responding rationally to changes in the task structure in order to improve their accuracy. However, using hoops of unequal size did not help participants to solve the focus-or-divide task optimally, as there is no systematic tendency to shift from centre to side positions as Δ increases. This replicates previous versions of this experiment (Clarke & Hunt, 2016; James et al., 2017; James et al., 2019).

**Fig. 4:**
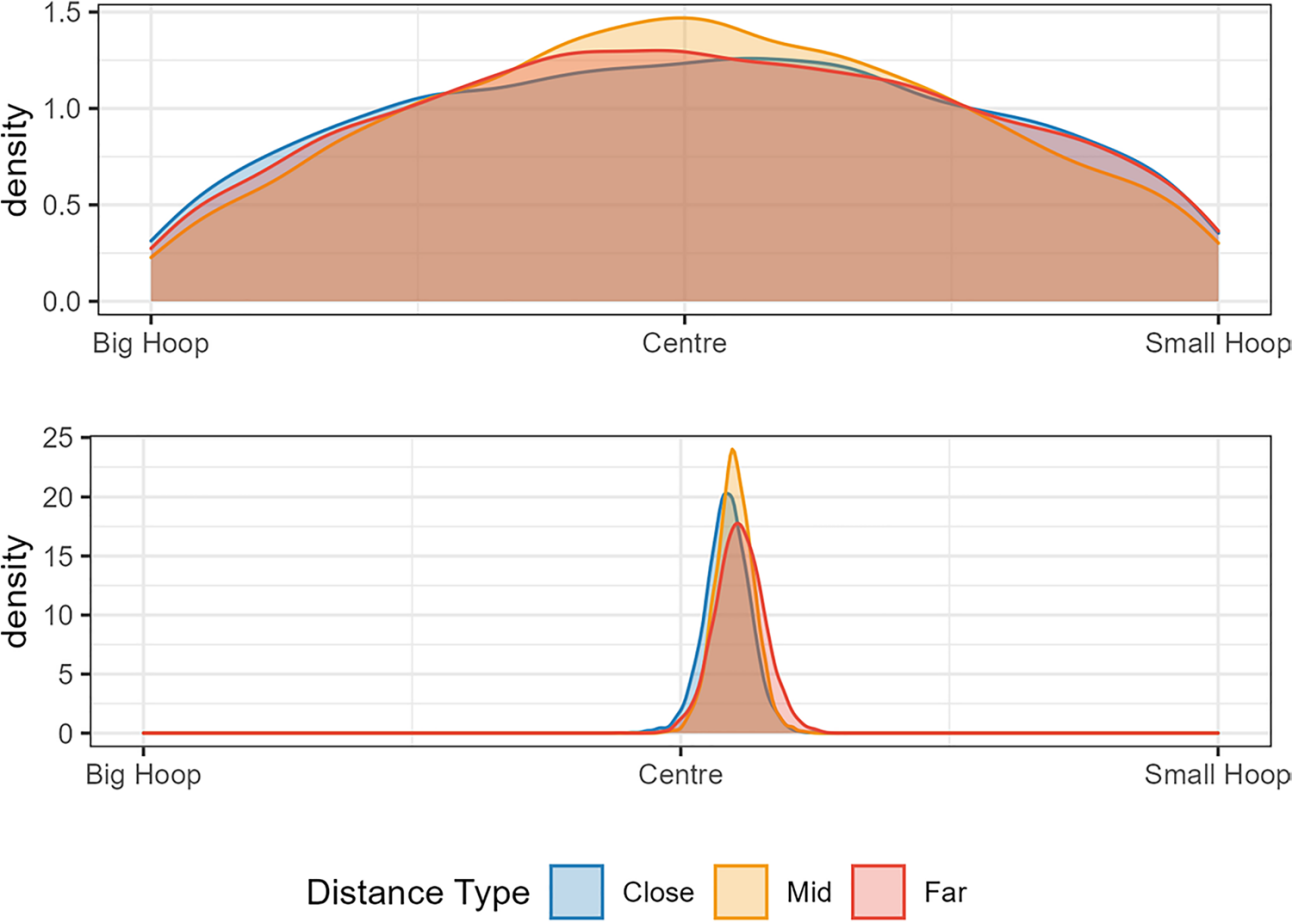
Bayesian model of Experiment 1 data. The top panel shows the *prior* predictions for standing position choices. The bottom panel shows the *posterior* predictions once the model had been conditioned on the data. The analysis confirms that standing position is not adjusted with the distance between the hoops (close, mid, far). (Colour figure online)

Standing position data were modelled using a Bayesian Beta regression. The data were transformed so that 0 < standing position < 1 in order to fit a Beta distribution. The data were coded so that ≈0 meant the participant stood next to the big hoop, 0.5 was the centre, and ≈1 reflected the small hoop. The priors for this model were relatively wide Student T distributions all centred on 0, indicating no bias in favour of either side and no effect of distance (Fig. 4, top panel). The model results suggested that participants, in general, had a bias towards standing closer to the small hoop (mean of 0.549, 95% HDPI of |0.508, 0.591|). We can be reasonably confident about this result because the probability that the mean is greater than 0.5 given the data is 98.8%. This can be seen in the posterior in the bottom panel of Fig. 4. Also, note that distance did not appear to have an effect on position, that is, participants were generally biased slightly towards the smaller hoop across all distances tested. This indicates that participants did not even approach an optimal strategy, and instead chose a place to stand that would (on average) somewhat balance their chance of success for each hoop, irrespective of the distance between them.

#### Accuracy

We use the performance data collected in Session 1 to calculate each participant’s optimal accuracy, by estimating what their throwing accuracy would have been had they chosen the optimal place to stand. To obtain a lower bound, we estimate each participant’s minimum expected accuracy based on a counter-optimal standing position. In this experiment, the counter-optimal strategy would be to stand next to one of the hoops when they are close together, and in the centre when they are far apart, as this would produce the lowest expected chance of success. Both of these measures vary across different participants, and the ranges are shown in Fig. 3b. We can compare these to the accuracy that would be expected given a participant’s actual standing position choices in Session 2, using their performance in Session 1. We present this measure instead of actual throwing accuracy in order to remove variability due to chance and variation in throwing accuracy from one trial to the next, and to provide an estimate that is more directly comparable to the estimates for optimal and counter-optimal accuracy.

From Fig. 3b, it is clear that none of the participants made use of the optimal strategy. Expected accuracy fell far short of optimal accuracy. This is expected from the standing position results, as the majority of participants did not modify their standing position with the distance between hoops. The decrease in accuracy as distance increases is consistent with previous studies and reflects the fact that the decisions have less effect on accuracy when the two tasks are easy.

### Discussion

The aim of this experiment was to provide participants with a concrete and intuitive difference between the two targets to use in guiding their decisions. We can conclude from this experiment that participants are sensitive to the differences in hoop size and adjust their behaviour to increase their expected accuracy. However, creating an asymmetrical decision did not help participants solve the focus-or-divide problem. Participants stood in a central location just as often when the two targets were close together as when they were far apart. This led to accuracy rates that fell far short of what they could have been had an optimal strategy been adopted.

We confirmed and extended this conclusion in an experiment presented in the Supplementary Material, using a different manipulation of the symmetry between the two possible targets and a different task context. Briefly, we used an eye-movement task and presented a small, brief target inside either a left or right square (see also Clarke & Hunt, 2016; James et al., 2019; Morvan & Maloney, 2012). Participants had to choose a place to fixate in anticipation of the target appearing. The distance between the squares determined whether the best location to fixate was between the two squares (when they were closer), or inside one of the two squares (when they were too far apart to be visible from the center). We manipulated the probability of the target appearing in one square over the other (with an 80/20 share across the two locations). The results are in line with the results we observed above: participants adjust their fixation decisions to match the probability manipulation, but do not make more optimal fixation decisions with respect to the distance between the squares.

## Experiment 2: Two throws

Even though there was a logical reason to favour one hoop over the other in Experiment 1, participants would still experience positive feedback (i.e., they would achieve the goal on that trial) when selecting the hoop that happened to be designated as the target on that trial, and negative feedback (i.e., they would miss) when selecting the nondesignated hoop. The sequence of which of the two hoops (North or South) was designated as the target was predetermined and unpredictable, so when participants did stand near one hoop, they could expect to have guessed correctly on about half the trials. Even though participants are informed that the sequence of north and south designations is random, and the experimenter is clearly reading it off a list, the participant might question whether the sequence is truly random, especially when streaks or other specious patterns happen to emerge (e.g., Nickerson, 2002). Searching for patterns is a cognitively demanding task in itself (Wolford et al., 2004), which may have distracted participants from making better focus-or-divide decisions. It is also possible that choosing to stand near what turns out to be the target or nontarget hoop on any given trial could influence decisions on subsequent trials, leading to less optimal choices, similar to those predicted by regret theory (Loomes & Sugden, 1982). In other words, choosing to stand near one hoop and having it turn out not to be the target could instill a negative emotional response, which would lower the expected utility of standing near either hoop relative to standing in the center.

In Experiment 2, participants completed two blocked conditions. One block was the same as the previous experiments, in which participants choose a place to stand and then are told which of the two hoops is their target on that trial. In the other block, instead of only one of the hoops becoming the target, participants threw two beanbags—one at each hoop—on every trial. In the nomenclature presented in the introduction: *P*_*c*_(*A*) = *P*_*c*_(*B*) = 1. This change to the experiment removes any reason for participants to try and “discover” an underlying pattern in the task, because there are no random variables except which beanbag color is drawn from the bag (and the participant does the drawing). Throwing at both hoops also removes any disappointment associated with selecting and standing near the “wrong” hoop. Importantly, to maximize accuracy to achieve both goals, the optimal strategy remains the same. Standing in the center when the hoops are close together will increase the likelihood that participants will hit both, and standing next to one when they are far apart will ensure that at least one of the goals will be achieved.

### Methods

#### Participants

Eighteen participants (eight male) took part in this experiment, with an average age of 22 years (between 19 and 30). Participants were recruited via word of mouth. None had previously participated in any related experiments.

#### Procedure

This experiment followed the same protocol as in Experiment 1, with the following exceptions. First, each hoop was the same size (0.4 m in diameter) so standing equidistant from both would give participants an equal chance at each target. The Supplementary Materials present individual throwing performance from Session 1, which was used to determine the hoop positions for each participant in Session 2, using the same methods described in Experiment 1. Session 2 was split into two blocked conditions: the one-throw and the two-throw condition. The order of these blocks was counterbalanced across participants. The one-throw condition followed the same procedure as in Experiment 1. In the two-throw condition, participants still selected one bean bag at a time from a bag containing nine, with three of each colour. They were then handed a second bean bag of the same colour from a separate pile. Participants were then, as in Experiment 1, instructed to choose somewhere to stand, at which point they would notify the experimenter. They would then throw each bean bag to each of the two hoops of the same colour, in whichever order they preferred. The stated goal for both conditions was to get as many bean bags into the hoops as possible. As before, the experimenter would record the standing position and throwing accuracy on each trial, and clear the beanbag from the paved area after each throw.

### Results

#### Standing position

**Fig. 5:**
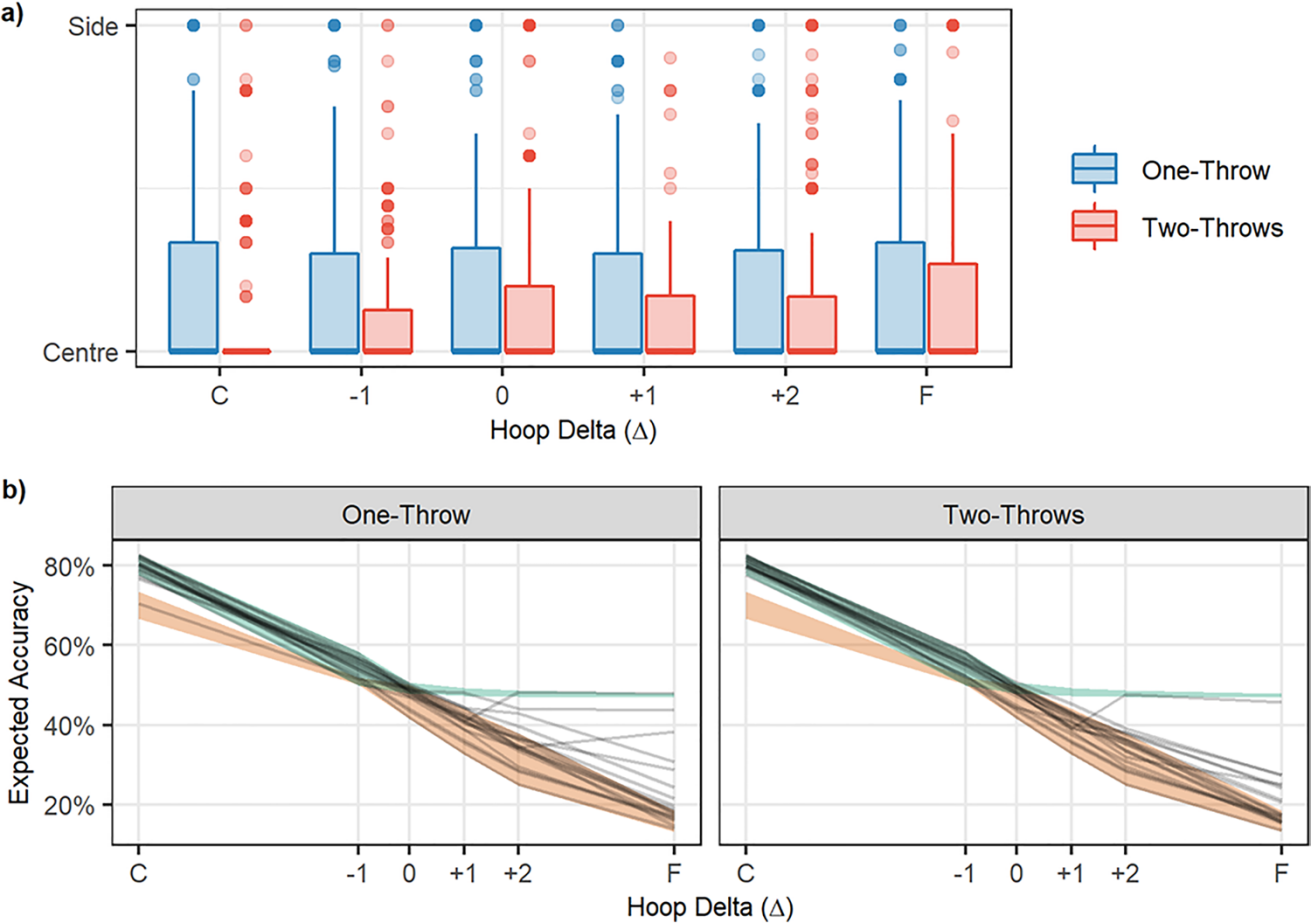
Results of Experiment 2. **a** The boxplots show the distribution of standing positions across the distances tested in this experiment. Please note that these boxplots are highly skewed in all cases; the median standing position was the center. **b** The black lines show the expected accuracy for each participant based on their standing positions across the distances tested. The shaded areas represent boundaries on accuracy, with green showing expected accuracy for optimal decisions and orange for counter-optimal decisions. (Colour figure online)

From Fig. 5a, it is clear that there was little change in standing position with increasing distance between hoops in either the one-throw or the two-throw condition. In the two-throw condition, participants opted more often to stand in the centre overall. This was particularly true for the closest hoop separation, but the standing position remained closer to center for the two-throw condition across all separations. The benefit of consistently standing near the center when the hoops are close together does not outweigh the cost of standing near the center when they are far apart. Consequently, participants (as a whole) did not perform the task in a more optimal way (see accuracy results below).

**Fig. 6:**
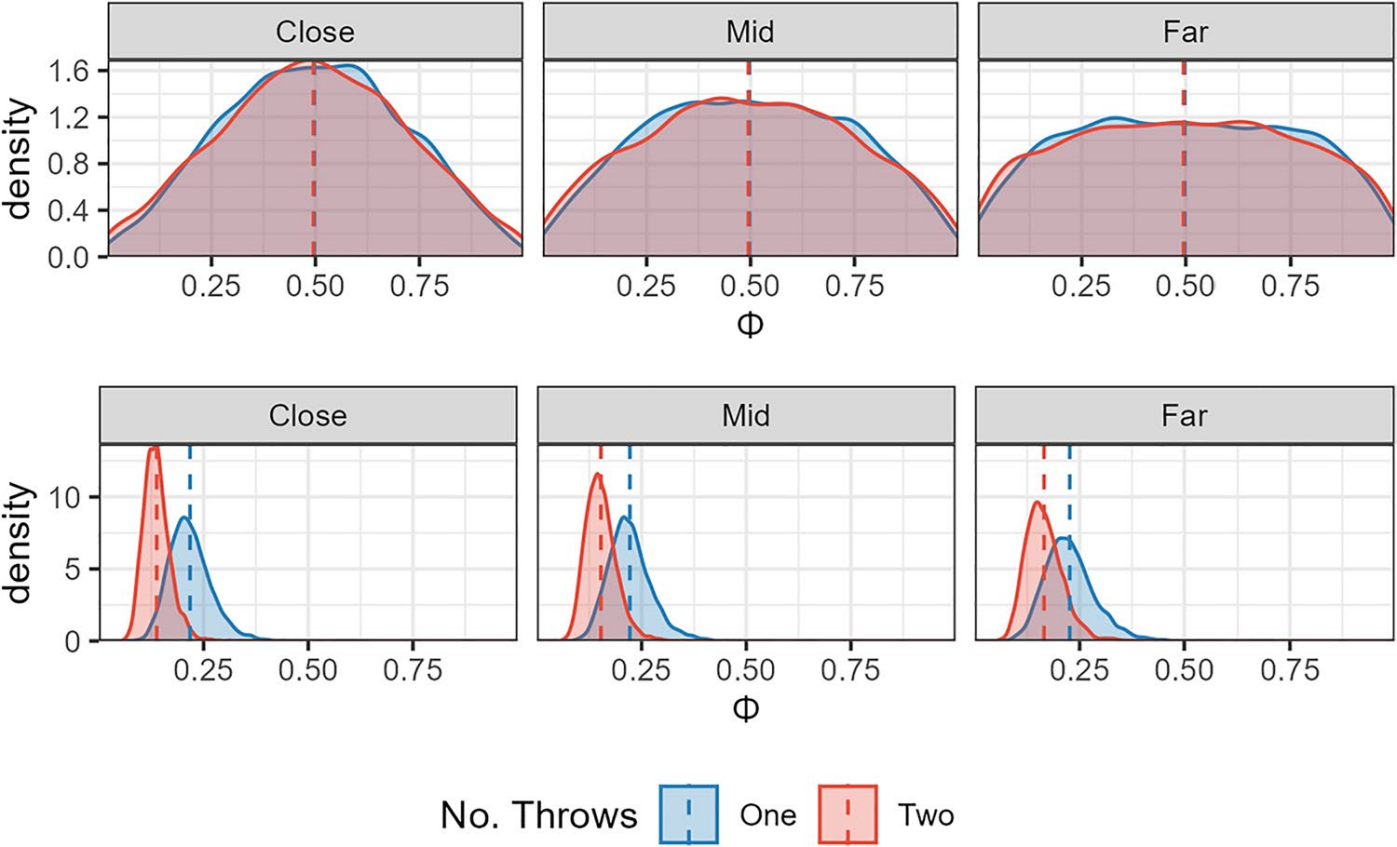
Bayesian model of Experiment 2 data. The top panel shows the prior predictions for the Two-Throw Experiment across three hoop separations (the three panels: close, mid, and far). The bottom panel shows the posterior predictions. The analysis confirms that standing position is similar across hoop separations for both the one-throw and the two-throw condition, and that throwing to both hoops is associated with standing closer to the middle

The model for this experiment was a beta regression with no intercept parameter. The analysis suggested that there was a greater tendency for participants to stand further from the centre in the one-throw condition (mean of 0.222, 95% HPDI of |0.126 , 0.313|) than in the two-throw condition (mean of 0.15, 95% HPDI of |0.083 , 0.229|) with P(Onethrow > Two-throw | data) = 91%. However, as can be seen from Fig. 6, the difference is generally small and consistent across all distances. This means that when participants were given the opportunity to throw to both hoops (i.e., in the two-throw condition), they were still suboptimal in their performance if not even less optimal as they generally opted for more central standing positions even in the most difficult setting.

#### Accuracy

Figure 5b shows that most participants’ accuracy (black lines) dropped below that which would be expected had they employed the optimal strategy (green). Note that, like for Experiment 1, we are calculating an estimate of accuracy based on the standing position choices and throwing performance of each individual, to remove variability and ease comparison to optimal and suboptimal baselines. One of the 18 participants was close to achieving optimal expected accuracy in both conditions. This is consistent with other results showing the occasional participant approaching an optimal strategy, but the majority performing far below this (e.g., in Clarke & Hunt, 2016, Experiment 2, one of the 12 participants approached optimal).

### Discussion

Most participants failed to perform optimally in the task, whether they had to throw a beanbag into one hoop, or both. The results rule out a search for patterns or choice regret as the reason for suboptimal decisions because the decisions are similarly sub-optimal in the absence of any uncertainty about which hoop would be the target. The results also suggest that the potential regret associated with selecting the wrong target (e.g., Loomes & Sugden, 1982) is not the reason participants stand in the center when they should be standing near one of the targets. Instead, we see participants stand even closer to the center in the two-throw condition, when the potential for this regret is no longer present. One potentially important feature of the two-throw condition is that moving to stand next to one hoop all but guarantees the participant will miss when they have to throw to the other one. The prospect of a certain miss may have outweighed the increased probability of success that would have been gained by moving closer to one hoop, consistent with the predictions of prospect theory (Kahneman & Tversky, 1979). That is, people appear to slightly prefer the prospect of probably missing both targets over definitely missing one and definitely hitting the other one. It is important to note that even though participants did keep closer to the center in the two-throw condition on average, they also selected a range of different standing positions in both conditions, which loss aversion cannot explain. In any case, the results rule out an explanation for poor prioritizing decisions based on people trying to predict which hoop would be the target, and/or being disappointed when they guessed incorrectly.

## Experiment 3: Unequal reward

The expected utility of a particular course of action is the sum of the expected gain or value of each possible outcome multiplied by the probability of each of these outcomes occurring. Thus far, the “gain” associated with each target was still symmetric: leaving aside the intrinsic reward of getting the beanbag in the hoop, in terms of monetary value, the two goals were equally null. In Experiment 3, we introduced monetary rewards for accurate performance, resulting in the new expectation:

3 $$E\left(\phi,\Delta\right)=R_AP_c\left(A)P_s(A\left|\phi,\Delta\right.\right)+R_BP_c(B)P_s\left(B\left|-\phi,\Delta\right.\right)$$

where *R*_*A*_ *and R*_*B*_ *are the rewards for achieving goals A and B*.

In the previous experiments using this paradigm, *R*_*A*_ = *R*_*B*_*.* In the current experiment, we asked participants to choose the relative value of the two targets. Before each trial, we asked them to choose either an equal split (*R*_*A*_ = *R*_*B*_), or to assign 80% of the reward to one target and 20% to the other (i.e., *R*_*A*_ = 0.8, *R*_*B*_ *=* 0.2). With this design, participants are offered the chance to avoid the Buridan’s Ass dilemma altogether, by ensuring that they are no longer equidistant from two equally rewarding options. This allows us to evaluate participants’ preference for symmetrical options, as well as the effect of asymmetrical values on their decisions (presuming they choose these).

Rewards have been shown to improve decisions in some contexts (e.g., Goodnow, 1955; Phillips & Edwards, 1966), although there are limits (for a review, see Camerer & Hogarth, 1999). In a gamified version of the focus-or-divide dilemma (James et al., 2019), in which a penguin character could earn fish rewards for accurate performance, participants improved their performance on the task relative to participants who were not given this additional motivation. However, the reason for the improvement was not participants making better prioritizing decisions. Rather, decisions with this additional motivation remained equivalently suboptimal, and instead participants performed better in other aspects of the task (such as making fewer key-press errors, and monitoring locations more vigilantly). Similarly, offering financial incentives for accuracy (Morvan & Maloney, 2012) did not improve decisions relative to not doing so (Clarke & Hunt, 2016). Overall, this suggests a lack of extrinsic reward is unlikely to explain the suboptimal decisions observed in the throwing experiments.

The current experiment goes beyond simply assessing the effect of rewards, by providing insight into how the Buridan’s Ass dilemma is regarded by participants. Before they made a decision about where to stand, they could designate one of the targets as more valuable than the other. The choice with the highest expected payoff, irrespective of distance between hoops, is to always choose an unequal split and then stand next to the hoop with the greater value. Therefore, in this experiment, not only do equally rewarding and equally difficult hoops create a Buridan’s Ass dilemma, they also decrease the potential winnings of participants. The conditions of this experiment enable participants to avoid this equivalent-options scenario entirely, by always splitting the reward unevenly. In so doing, participants would also be adopting a more financially rewarding strategy.

A related question is whether consistent relationships will emerge between hoop distance, people’s choices of how to split the reward, and their choices in where to stand. The requirement to judge how to divide the reward might nudge people to think about the consequence of the hoop separation more carefully. As the hoop separation increases, they may consider splitting the reward unevenly, because they recognize that they are likely to fail from using a central strategy at far hoop separations. Thus, there may be a tendency, at least among some participants, to divide the reward unevenly at larger hoop separations. Among these participants, they may also commit to standing closer to the hoop they have made more valuable. Indirectly, this could lead participants who split the reward unevenly to approach an optimal strategy.

### Methods

#### Participants

Twenty participants took part in this experiment (15 female) with an average age of 22.6 years (between 20 and 30). All participants were recruited via word of mouth at the University of Aberdeen. The power analysis was the same as for the previous experiment.

#### Procedure

Participants signed a consent form which contained details about the reward schedule and how much they could expect to earn on average. All participants were given £4 as a baseline and were told that they could expect to earn an additional amount ranging from £0 to £4.80 depending on their performance. They were also told that we expected them to earn between £1.50 and £2.50 on average.

This experiment followed a similar procedure to that of Experiments 1 and 2. However, in this experiment, both the measuring and decision sessions took place in one session. First, participants were taken to the paved area in order to measure their throwing ability across the same eight distances used for the small hoops in Experiment 1 (slabs 3 to 19 slabs or 1.38 m to 8.74 m), with participants throwing 12 bean bags for each distance (96 trials total). After this, they performed a brief computer-based task in the lab. The task was a brief pilot of an unrelated experiment, which involved detecting shapes among cluttered and uncluttered scenes. This was done only to make efficient use of participants’ time while the experimenter calculated their performance curves and set up the hoops for the second session, much in the same way as before. For the final task, participants were taken back to the paved area to complete the decision session, as follows.

There were two main changes to the paradigm. First, four hoop distances were used: the distances at which participants were 90%, 75%, 25%, and 10% accurate, based on their individual Session 1 performance. Each of these distances was tested 3 times for a total of 12 trials. Second, participants were told they had 50p to split between the two target items in one of two ways. They could either split it equally across both potential targets (25p/25p) or make it an 80/20 split (40p/10p). Participants were asked how they would like to split the money before they made a choice about where to stand. If they opted to make an unequal split, they were asked which hoop they would like to be worth 40p and which 10p. Participants were informed that the target hoop had been randomly predetermined so that each hoop was equally likely to be the target on each trial. It was reaffirmed that any money they earned by successfully throwing the bean bag into the target hoop would be given to them upon completing the experiment. Participants would then pick a place to stand, at which point they would be told which hoop was the target for that trial. The experimenter recorded standing position and throwing accuracy and cleared each beanbag from the area after each throw.

### Results

#### Splitting the reward

**Fig. 7:**
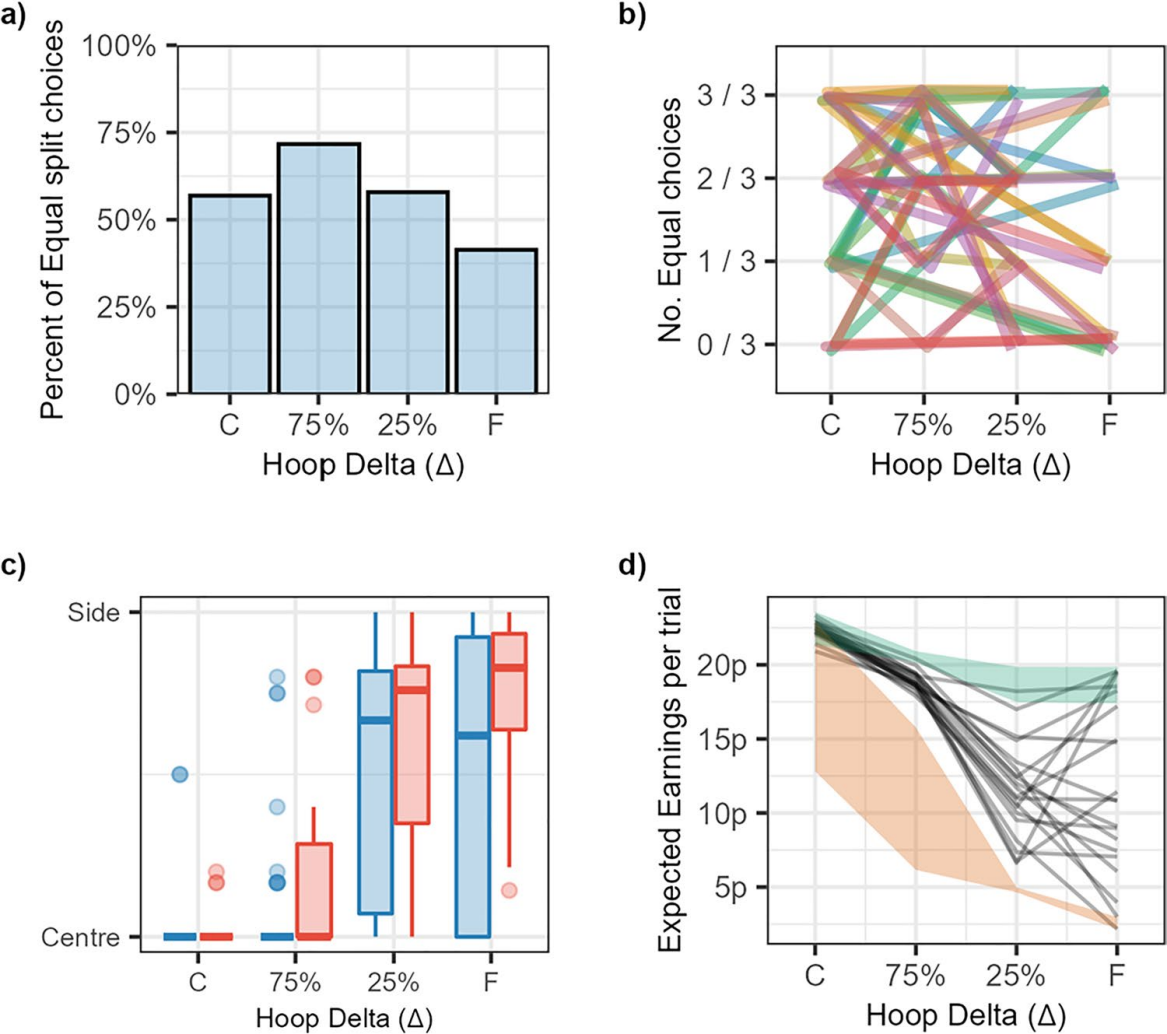
Results from Experiment 3*. ***a** The proportion of times that participants opted for an equal split across the different distances. **b** This panel shows the same data as in panel **a**, but each participant has a unique line to trace their decisions across each distance. **c** Boxplots indicating the standing positions for the four distances tested with the colours (red for unequal and blue for equal) showing whether participants had opted for an equal or unequal split. **d** This panel shows the average expected earnings across the different distances tested. The shaded areas represent the ranges for the greatest expected outcome (green) and the worst (orange). (Colour figure online)

We can see from Fig. 7a that participants selected the 50/50 and 80/20 reward splits about equally often, with a slight favouring of the equal split. There is also an increased tendency towards the unequal split as Δ increases, but even at the farthest hoop distance, participants choose equal splits around 40% of the time. Figure 7b shows individual choices of how to split the reward. From this figure we can see that it is not that some participants choose uneven splits and some choose even splits; rather, all participants choose uneven on some trials and even splits on others. Five participants opted for an unequal split most of the time, but no participants followed the optimal rule of always splitting the value unequally and standing next to the more valuable target.

#### Standing position

**Fig. 8:**
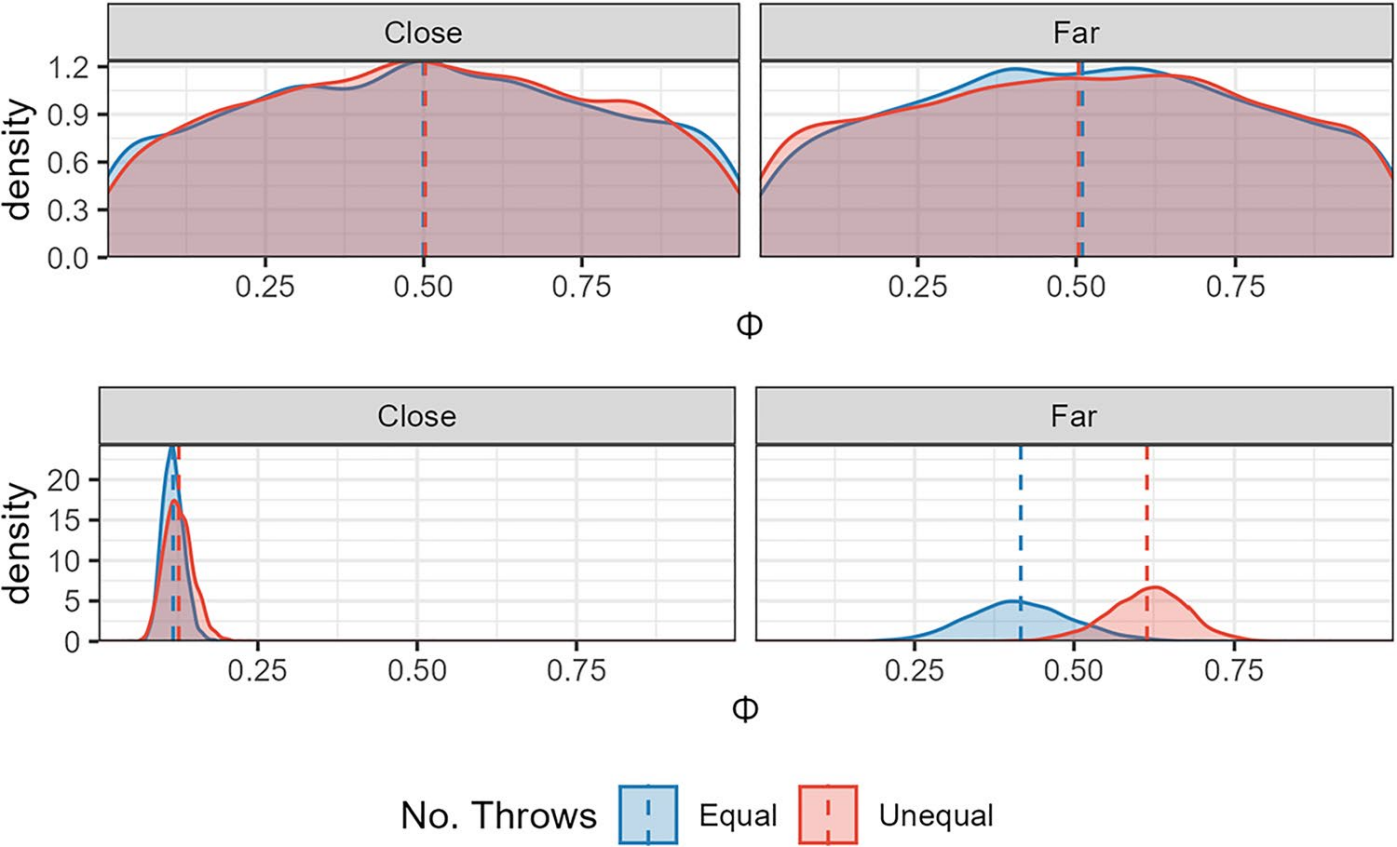
Bayesian model of Experiment 3 data. The top two plots show the predictions for the model that only sampled from the prior. The bottom plots show the results when the model had been conditioned on the data, split into the two closer hoop conditions (where they should stand in the center) and the two farther hoop conditions (where they should stand next to one)

Standing position choices are shown in Fig. 7c. When compared with the results of Experiments 1 and 2 (Figs. 3a and 4a), standing position choices in this experiment are more sensitive to Δ. This sensitivity was especially pronounced when participants had opted for an unequal split, shown in the red box plots. As in the previous analyses, we applied a Bayesian regression model using a Beta family of distributions with no intercept parameter. The predicted value (standing position) was normalized so 0 would equate to a participant standing in the centre, and 1 next to one of the target hoops. Due to the use of the Beta family, trials in which participants stood outside the range (of which there were six in total) were excluded from the analysis. The predictors of interest were split type (i.e., equal or unequal), and target separation. Target separation (Delta) was scaled so 1 represented the furthest separation for each individual participant. The output of this model can be seen in Fig. 8. The model suggested that participants were more likely to stand closer to one of the two hoops when the hoops were far apart. This confirms the descriptive results suggesting that participants made some use of the distance information available to them to adjust their standing position, unlike the previous two experiments.

This adjustment in standing position with hoop distance was more pronounced for trials in which participants had opted to split the reward unequally across the two options. This resulted in participants standing closer to the side hoop in for unequal trials (mean of 0.613, 95% HPDI of |0.488, 0.73|) than when they had opted for an equal split (mean of 0.417, 95% HPDI of |0.25, 0.582|) with the P(Unequal > Equal | data) = 95.7%. Although this goes in the direction that participants were more optimal in the presence of unequal financial rewards, they did not follow the truly optimal strategy as the truly optimal strategy was to split the money unequally on every trial and stand next to the target worth the larger amount.

#### Expected earnings

Figure 7d presents the expected earnings per trial. This value represents the expected earnings based on Session 1 throwing performance and the chosen standing positions, in order to ease comparison with the *optimal expected earnings:* this is what participants would have earned if they had adopted the optimal strategy of splitting the money unequally and then standing close to the more valuable hoop. Most participants fall far short of this. The lower bound is the counter-optimal earnings: This is what participants would have earned if they had made the poorest possible choices. The poorest possible choice would be to split the monetary reward unequally and then standing next to the lower value option for all distances except the furthest, in which case, standing in the centre was the worst choice for participants.

### Discussion

The optimal strategy in this task was to always split the reward unevenly and then stand next to the hoop with greater value at all hoop separations. However, participants slightly favoured the even split. This even-split choice had two negative consequences; first, the possible gains were smaller, particularly when the hoop separation was large. Second, when they chose the even split, participants were more likely to stand closer to the center when the hoop separation was large, relative to when they chose an uneven split. The bias towards the even split despite its negative consequences for financial reward is clear evidence that participants do not avoid Buridan’s Ass dilemmas when given the opportunity.

An additional interesting observation from this experiment was that standing position was modified by Δ more in this experiment than had been observed in the previous ones, suggesting that reward does facilitate optimal decisions in a focus-or-divide dilemma. However, reward for successful performance is only part of the story. On trials in which participants chose to split the reward unequally, they were more likely to choose standing positions that were closer to one of the potential target hoops than had they opted for an equal split. Therefore, participants needed not only a reward, but also the option to split the money unequally, before they were willing to move away from one target when the two targets were too far away to achieve success from the center. We can therefore conclude that the introduction of a monetary incentive to prioritize one target over the other was indeed effective in causing some participants to vary their strategy with distance more consistently. Given this, it is striking that participants persist in splitting the reward equally on the majority of trials. Even when the two targets were at the furthest separation, participants chose an equal split on over a third of the trials, leading to losses in potential monetary gains. Not only did participants stand to gain less money if they were successful when they had split the money equally, but participants were also less likely to be successful when they split the money equally, because they were less likely to adopt the optimal strategy of standing next to one hoop.

## General discussion

The current study rules out the Buridan’s Ass dilemma as a potential cause of poor resource-allocation decisions shown in previous studies (Clarke & Hunt, 2016; James et al., 2017; James et al., 2019;Morvan & Maloney, 2012). We had speculated that participants’ ability to adjust behaviour *ɸ* to maximize expected accuracy *E(ɸ,* Δ*)* might depend on having clearly different expected utility levels for tasks A and B for a given *ɸ*. That is, for this decision problem:

4 $$E\left(\phi, \Delta \right)={P}_c(A)x\ {P}_s\left(A\left|\phi, \Delta \right.\right)+{P}_c(B)x\ {P}_s\left(B\left|-\phi, \Delta \right.\right)$$

The hypothesis was that participants may be able to adjust *ɸ* to maximize *E* only when *P*_*s*_*(A) ≠ P*_*s*_*(B)*. We found consistent evidence against this hypothesis, that is, poor focus-or-divide decisions persisted even when A and B were unequal. In exploring this question, we extended the previous findings of suboptimal decisions to include conditions where the decision between the two goals is no longer arbitrary, or even necessary. Moreover, participants do not consistently take advantage of opportunities to avoid choosing between equal options, even when they could benefit financially from doing so.

In all three experiments, we replicated previous experiments showing that participants do not approach an optimal strategy in a focus-or-divide dilemma, and as a result, achieve throwing accuracy far lower than could have been achieved if they had. In Experiment 1 of this series, one of the two target hoops was smaller than the other. Participants tended to stand closer to the smaller hoop than the larger one, demonstrating that they are sufficiently motivated to use hoop size information to boost their chances of success, and capable of implementing an effective strategy to do so (similar to optimal spatial averaging in visually-guided reaching, reported by Chapman et al., 2010; see also Hesse et al., 2020). Nonetheless, participants were not able to maximize their chances of success by modifying their standing position decisions as the distance between the hoops changed. This resulted in accuracy that was far worse than could have been achieved, particularly when the hoops were far apart and participants persistently stood close to the center, causing their throwing accuracy to fall far short of 50%. These results show that giving participants a reason to prioritize one target over another did not lead to more optimal focus-or-divide decisions. A similar experiment in the supplementary material manipulated probability and showed a similar pattern, with participants usually (but not always) prioritizing the more likely target. These results support the notion that people are sensitive to information about the expected utility of different options and make use of it when making decisions (Gao & Corter, 2015; Wolford et al., 2004; Yellott, 1969). This is further evidence against the argument that perhaps participants are not sufficiently motivated to maximize success in this task; they are clearly monitoring their environment and exploiting opportunities to improve success rates where they perceive them. This aligns with previous results showing gamification of a focus-or-divide task, while improving other aspects of performance, does not bring participants any closer to optimal decisions about whether to stay in the center or shift to one target (James et al., 2019).

Experiment 2 included a condition where participants had to throw two beanbags, one at each hoop. In the two-throw conditions, participants no longer had to guess which hoop was likely to be the target; they knew both were targets. We reasoned that the two-throw condition should improve accuracy relative to the one-throw condition, if uncertainty about which hoop would be the target and the regret associated with selecting the “wrong” hoop had been dissuading people from implementing the optimal strategy. Inconsistent with this prediction, the mean throwing accuracy for the two hoops that would be expected based on where participants chose to stand in the farthest hoop separation condition was 22% and 21% in the one-throw and two-throw conditions, respectively. Not only are these values similar, they are far lower than the 50% accuracy that would be expected if participants had chosen to stand close to one hoop when they were far apart. We conclude that suboptimal choices are not due to the effects of trying to predict random sequences of targets, or to the regret associated with these predictions. This is not to say that under the one-throw condition participants do not try to find patterns in sequences of targets, or that they do not feel disappointment when they select the wrong hoop. Indeed, many of our participants verbally report engaging in prediction and experiencing regret when they are wrong. There are also certainly many circumstances in which trying to discern patterns in random sequences can lead to suboptimal choices, such as in the classic probability matching bias (e.g., Gaissmaier & Schooler, 2008), which we also observed in the experiment presented in supplementary materials. As noted in the introduction, the negative experience of regret can account for deviations from predictions of choice based on classic definitions of expected utility (Loomes & Sugden, 1982). In this focus-or-divide decision problem, however, the poor prioritizing choices persist even in conditions where explanations based on prediction and regret are no longer viable.

In Experiment 3, participants were given the choice to split a monetary award equally or unequally across the two hoops. It was on the trials where they split the reward unevenly that participants tended to vary their strategy more appropriately with task difficulty. This suggests that the interaction between asymmetry and financial reward facilitates the use of a more optimal strategy. Interestingly, however, none of our participants managed to consistently follow the optimal strategy of opting for an unequal split, then standing next to the most valuable target. Indeed, overall, they slightly preferred to evenly split the reward, choosing to do so on over half of trials overall. This result suggests that unequal rewards *can* lead to better focus-or-divide decisions^3^. The fact that participants did not seem to recognize that unequal rewards had this double benefit of both directly increasing potential winnings and indirectly facilitating more optimal decisions reinforces the previous findings that participants’ insight into the relatively simple logic of the optimal strategy is limited (Hunt et al., 2019). Participants are not easily “nudged” into better performance.

One caveat about the final experiment is that it is possible we sampled a group of participants who were more likely to make better focus-or-divide decisions in the first place. In previous experiments, a handful of individuals do approach optimal strategies in this task, so having more of these participants in the sample could lead to the impression that the conditions improved their performance, rather than having been better in the first place, which is why we have made within-group comparisons wherever possible in these experiments. More generally, individual differences in decision strategies are common, and present an important challenge for explaining the biases and heuristics people tend to use (e.g., Clarke et al., 2019; Jasper et al., 2017; Zhang et al., 2012). In our particular decision problem, the solution is trivially easy to implement when it is known (Hunt et al., 2019), so it is important to ensure the participants have not been exposed to the decision problem before. Our participants were naive insofar as they had not participated in any of our previous decision experiments, but we are sampling from a population of undergraduates who all complete psychology experiments regularly, and may communicate with one another about different experiments they have participated in. An experiment which involves throwing beanbags at hoops may stand out among the others, which tend to involve computer-based tasks and questionnaires, so the population we sampled from might be less naïve than the general population. Given that this was the last in a series of similar experiments, this population may have had more knowledge of the optimal solution than was the case earlier for the previous experiments. Another potentially important distinction between Experiment 3 and the previous two is that participants completed the two sessions of the experiment (measuring throwing accuracy task and the decision session) on the same day, while in Experiments 1 and 2 they were separated by a week. It could be that the fresher memory of throwing performance facilitated better use of that information in making decisions. That said, the fact that the participants made better decisions overall in this task does not undermine the conclusions that participants chose to divide the reward unequally relatively less often than equally, despite this leading to lower expected payoffs as well as worse decision-making.

The current results have some common ground with previous findings suggesting that people avoid situations that have the potential to incur a sure loss (Chapman et al., 2010; Hudson et al., 2007; Kahneman & Tversky, 1979). That is, participants may prefer equally poor chances across both targets over certain success for one and certain failure for the other. This preference may contribute to some participants’ choices, but it cannot be the entire explanation for poor decisions in the focus-or-divide dilemma, because if this were the case, participants would consistently “divide” instead of focus—in the beanbag throwing task, this would lead participants to consistently stand at, or at least near, the center. This is not the pattern that is observed in studies so far, including the experiments in the present series—some participants do stand in the center consistently, but just as many shift towards one target or another (see Supplementary Material to see the full range of individual decisions), and most make a wide range of different decisions about where to stand from trial to trial. The main consistent finding across experiments is that participants do not adjust their strategy as the distance between the hoops increases, but the behaviours they adopt instead of this adjustment are widely varied and cannot be accounted for solely by a bias to equate the odds of success across both hoops.

The results of this series of experiments also shed new light on how equivalent alternatives influence choice. In the introduction, we noted that some dynamic accumulator models assume options are evaluated independently, and the random noise in the accumulation of evidence about each option means that rarely are decision thresholds for different options crossed at the same time. In more general terms, the Buridan’s Ass dilemma can be easily resolved by random variation in attention or the relative salience of different features of the options (e.g., Bordalo et al., 2012). Alternatively, direct competition between different options (e.g., Teodorescu & Usher, 2013) can lead to protracted deliberation when the options are the same. Here, we tested the hypothesis that the presence of equal options might globally disrupt decision strategies. If so, competition between options could explain the poor prioritizing decisions observed previously, in which options were equivalent. There was very little evidence to support this hypothesis. We cannot interpret this as direct evidence against competition between options, because the competition might delay choice, while not affecting global strategy. We did not measure decision time in these experiments, nor would it be straightforward to do so. We can, however, rule out the possibility that competition between equivalent options can explain the general failure to solve the focus-or-divide dilemma observed across a large set of conditions. The results also clearly demonstrate that people do not avoid Buridan’s ass dilemmas when given the opportunity. In fact, they seem to prefer them over unequal options.

Decisions about how to prioritize tasks and goals are a common feature of daily life, and can carry large consequences, such as deciding how to invest limited time, money, and effort when faced with multiple options. Previous research has shown a persistent failure to prioritize tasks in a way that reflects the limits of our resources across a broad set of different task settings, including throwing and memory tasks (Clarke & Hunt, 2016), eye movements and visual detection (James et al., 2019; Morvan & Maloney, 2012) and visually guided reaching (Hesse et al., 2020). The current study extends this observation of poor prioritization even further, to conditions where the need to make arbitrary choices between options is no longer present. It seems likely, but remains to be established, whether this clear tendency to make suboptimal prioritization decisions in controlled laboratory conditions has measurable real-world consequences. If so, simple interventions to improve these decisions could have wide-ranging implications.

## Supplementary information

ESM 1 (PDF 1091 kb)

## Footnotes

1 This requires a few assumptions about the nature of *f*(Δ) = *P*_*s*_*(A|ɸ* = 0*,* Δ*)*. For example, when Δ is small, the centre strategy is optimal if *f*(Δ − *ɸ*) + *f*(Δ + *ɸ*) < 2*f*(Δ). When Δ is large, we require f(0) + *f*(2Δ) < *f*(*ɸ*) + *f*(2Δ − *ɸ*). A formal description of the family of functions for which this holds is outside the scope of this paper. We have verified that these criteria hold for the vast majority of empirical psychometric curves collected during our experiments.

2 The week’s delay was for logistical reasons; a group of seven undergraduate students helped with data collection as part of a research methods class, and the week delay between sessions simplified coordination of testing.

3 We do not know, nor is it within the scope of the current investigation, whether the agency of the participant in allocating the reward to the targets is a necessary condition of this improvement. It is an interesting and open question whether this improvement would still be observed if it was the experimenter who assigned monetary values to the targets.
