# Supplementary material for: Six of one, half dozen of the other: Suboptimal prioritizing for equal and unequal alternatives
Source: Mem Cognit. 2022 Oct 12;51(2):486–503. doi: 10.3758/s13421-022-01356-5 (PMC9950175; doi:10.3758/s13421-022-01356-5)
Supplement: Supplementary file 1 — (PDF 1091 kb) [file 13421_2022_1356_MOESM1_ESM.pdf]

Supplementary Material For Asymmetry paper

Contents

1 Session Info **1**

2 Power Analysis **3**

3 Additional Analyses **4**

3.1 Experiment 1: Hoop Size 4

3.2 Experiment 2: Two Throws 8

3.3 Experiment 3: Reward 11

4 Probability Matching Experiment **14**

4.1 Note 14

4.2 Introduction 14

4.3 Methods 15

4.4 Results 19

4.5 Discussion 22

Bibliography **23**

These supplementary materials present full details of all R packages used in the analysis; a detailed explanation of our simulation-based power analysis; and Bayesian models of the results discussed in the main manuscript. In addition, we also present details a further, related experiment using eye-tracking and a probability matching paradigm.

Please see the accompanying source Rmd file for the code.

1 Session Info

```
sessionInfo()
```

```
## R version 4.0.4 (2021-02-15)
## Platform: x86_64-w64-mingw32/x64 (64-bit)
## Running under: Windows 10 x64 (build 19042)
##
## Matrix products: default
##
## locale:
```

```
## [1] LC_COLLATE=English_United Kingdom.1252
## [2] LC_CTYPE=English_United Kingdom.1252
## [3] LC_MONETARY=English_United Kingdom.1252
## [4] LC_NUMERIC=C
## [5] LC_TIME=English_United Kingdom.1252
##
## attached base packages:
## [1] grid      stats     graphics  grDevices utils     datasets  methods
## [8] base
##
## other attached packages:
##  [1] psyphy_0.2-2    gridExtra_2.3   png_0.1-7       brms_2.15.0
##  [5] Rcpp_1.0.7      forcats_0.5.1   stringr_1.4.0   dplyr_1.0.6
##  [9] purrr_0.3.4     readr_2.0.0     tidyr_1.1.3     tibble_3.1.2
## [13] ggplot2_3.3.5   tidyverse_1.3.1 tidybayes_2.3.1
##
## loaded via a namespace (and not attached):
##   [1] minqa_1.2.4          colorspace_2.0-1     ellipsis_0.3.2
##   [4] ggridges_0.5.3       rsconnect_0.8.18     markdown_1.1
##   [7] base64enc_0.1-3      fs_1.5.0             rstudioapi_0.13
##  [10] rstan_2.26.1         farver_2.1.0         svUnit_1.0.6
##  [13] DT_0.18              fansi_0.5.0          mvtnorm_1.1-2
##  [16] lubridate_1.7.10     xml2_1.3.2           codetools_0.2-18
##  [19] bridgesampling_1.1-2 splines_4.0.4        knitr_1.33
##  [22] shinythemes_1.2.0    bayesplot_1.8.1      projpred_2.0.2
##  [25] jsonlite_1.7.2       nloptr_1.2.2.2       broom_0.7.8
##  [28] dbplyr_2.1.1         ggdist_3.0.0         shiny_1.6.0
##  [31] compiler_4.0.4       httr_1.4.2           backports_1.2.1
##  [34] assertthat_0.2.1     Matrix_1.3-2         fastmap_1.1.0
##  [37] cli_3.0.1            later_1.2.0          prettyunits_1.1.1
##  [40] htmltools_0.5.1.1    tools_4.0.4          igraph_1.2.6
##  [43] coda_0.19-4          gtable_0.3.0         glue_1.4.2
##  [46] reshape2_1.4.4       V8_3.4.2             cellranger_1.1.0
##  [49] vctrs_0.3.8          nlme_3.1-152         crosstalk_1.1.1
##  [52] xfun_0.24            ps_1.6.0             lme4_1.1-27.1
##  [55] rvest_1.0.0          miniUI_0.1.1.1       mime_0.11
##  [58] lifecycle_1.0.0      gtools_3.9.2         MASS_7.3-54
##  [61] zoo_1.8-9            scales_1.1.1         colourpicker_1.1.0
##  [64] hms_1.1.0            promises_1.2.0.1     Brobdingnag_1.2-6
##  [67] parallel_4.0.4       inline_0.3.19        shinystan_2.5.0
##  [70] curl_4.3.1           gamm4_0.2-6          yaml_2.2.1
##  [73] StanHeaders_2.26.1   loo_2.4.1            stringi_1.7.3
##  [76] dygraphs_1.1.1.6     pkgbuild_1.2.0       boot_1.3-28
##  [79] rlang_0.4.11         pkgconfig_2.0.3      matrixStats_0.59.0
##  [82] distributional_0.2.2 evaluate_0.14        lattice_0.20-41
##  [85] rstantools_2.1.1     htmlwidgets_1.5.3    processx_3.5.2
##  [88] tidyselect_1.1.1     plyr_1.8.6           magrittr_2.0.1
##  [91] bookdown_0.22        R6_2.5.0             generics_0.1.0
##  [94] DBI_1.1.1            pillar_1.6.1         haven_2.4.1
##  [97] withr_2.4.2          mgcv_1.8-36          xts_0.12.1
## [100] abind_1.4-5          modelr_0.1.8         crayon_1.4.1
## [103] arrayhelpers_1.1-0   utf8_1.2.1           tzdb_0.1.2
## [106] rmarkdown_2.9        readxl_1.3.1         callr_3.7.0
## [109] threejs_0.3.3        reprex_2.0.0         digest_0.6.27
```

```
## [112] xtable_1.8-4         httpuv_1.6.1         RcppParallel_5.1.4
## [115] stats4_4.0.4         munsell_0.5.0        shinyjs_2.0.0
```

2 Power Analysis

Our power analysis made use of simulations and resampling approaches based on data that we previously collected. Resampling was carried out in order to establish if adding more participants to our sample sizes would have lead to a reduction in uncertainty around our estimates. The experiments were all interested in the extent to which a distribution of standing position shifted with the introduction of a new factor. As such, one power analysis was carried out to determine what sort of sample size would be sufficient to detect a small shift in the mean of this distribution.

We started by fitting a a beta distribution to each participant's data from the *Throwing Experiment* in Clarke and Hunt (2016) using the `fitdistrplus` package (as can be seen in Figure 1. In the original data, normalised standing position was coded $\phi \in [0, 1]$ with 0 indicating a central standing position, and 1 indicating the participant stood by either of the two hoops. As we are *breaking the symmetry* between the two targets in this new experiment, we will now treat standing position as $\phi \in (0, 1)$ with 0 and 1 representing the positions of the large and small hoop respectively. The central midpoint is now represented as $\phi = 0.5$.

This is directly linked to the idea behind the Hoop Size experiment in which we aimed to observe whether participants would shift towards one side when presented with hoops of different sizes. However, this can be applied to the other experiments in that they are also looking at how the distribution shifts given a new factor in the experiment.

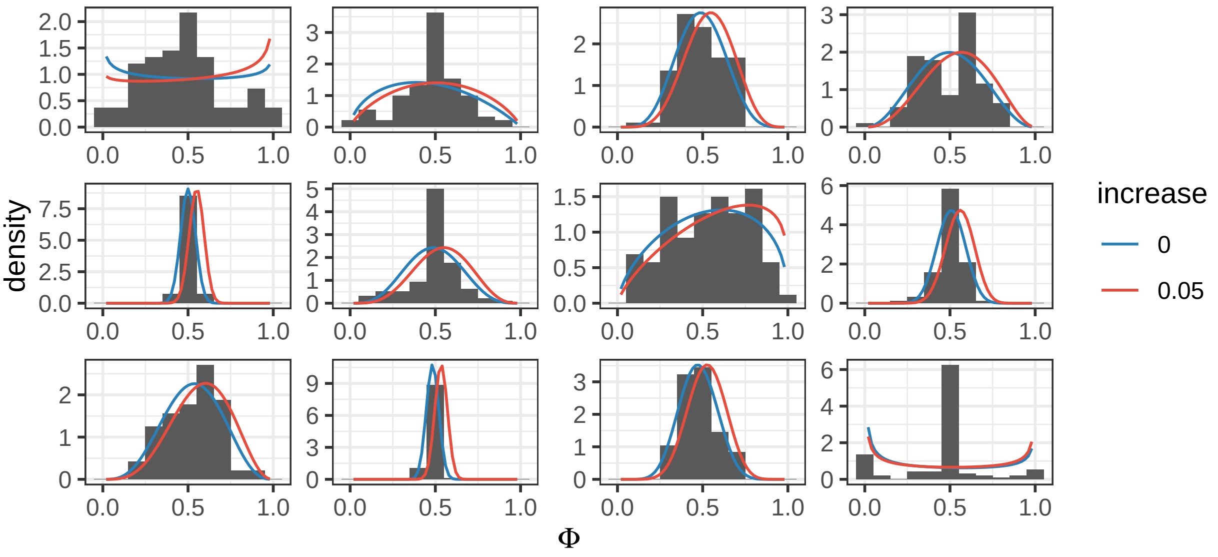

Figure 1: These plots show the various distributions that were fit to each participant. These were then used to draw random samples from in order to simulate participants shifting towards one target.

The fit with the empirical data is reasonable, although we under-estimate the frequency of standing positions $\phi \approx 0.5$. In this case 0.5 is considered the central point with 0 and 1 representing the left and right hoop respectively. We assumed that by introducing a difference in size between the two hoops that this distribution would shift towards the smaller hoop so as to balance their chances of success. We assumed that the difference in hoop size would cause participants to stand slightly closer to the small hoop, in this case the shift in mean would be +0.05.

We can now these use distributions to simulate experiments with $N = 3 \ldots 24$ participants and 72 trials. Figure 2 shows the uncertainty surrounding the mean estimate for the smallest difference tested (5%). After 15 participants, the uncertainty surrounding the estimate appears to plateau which demonstrates that the sample size of 21 was sufficient to detect an effect of this size.

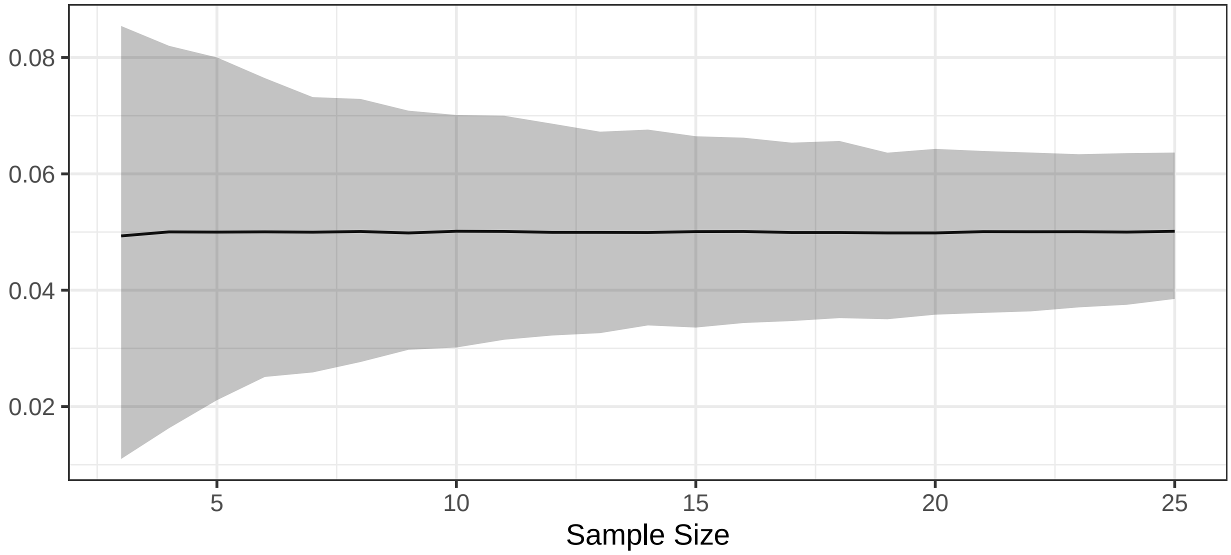

Figure 2: This figure shows how the 95% HDI around the mean difference for the smallest difference simulated (5%) changed with a larger sample size

3 Additional Analyses

In the analyses below, we use multi-level Bayesian beta regression to model the change in standing position ϕ when we 1) use unequal hoop sizes, 2) remove the need to chose between hoops, and 3) provide unequal rewards.

3.1 Experiment 1: Hoop Size

Figure 4 shows the data for each or the participants. Each plot shows the standing position on every trial for each of the standardised distances the participants were tested at. The dots are set to be somewhat translucent so darker regions demonstrate the participant stood in this location more often. Anything above the dashed line demonstrates the participant shifting towards the smaller hoop, while below represents the participant opting to be closer to the big hoop.

The data from this experiment were analysed using a Bayesian beta regression. The recorded data for standing positions were transformed to be between 0 and 1, with 0 representing the larger hoop and 1 representing the smaller hoop. Therefore, the central point would be 0.5, and meaning anything above this value would demonstrate a shift in participant's behavior away from the mid-point and towards the small hoop. The model included normalised hoop delta as a predictor to see how participants changed position with an increasing distance. This was also entered as a random effect by participant.

3.1.1 Modelling

For all models, we opted to use priors that were weakly informative. The chosen priors made the weak assumption that there was no effect of the experimental manipulations introduced in this series of experiments.

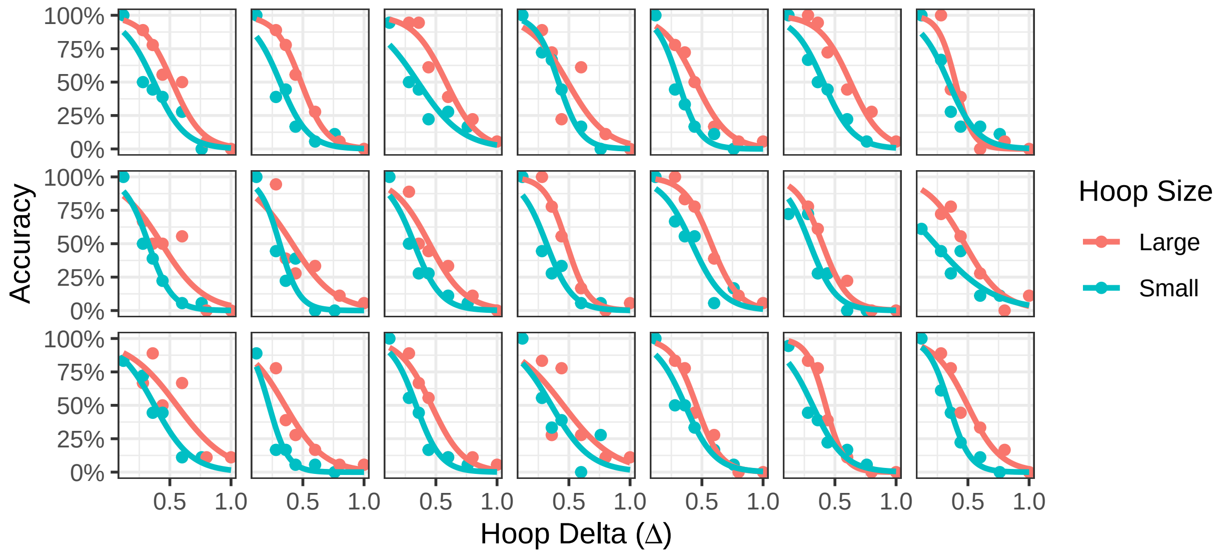

Figure 3: Each facet shows how a participant's accuracy dropped as a function of distance for both hoop sizes that were used in the experiment. The x-axis has been normalised so that 1 represents the furthest distance tested.

The models themselves looked as follows with the prior predictive checks having the additional "sample_prior" argument set to "*only*":

```
# run model
Hoop_size_m1 <- brm(norm_dist2 ~ norm_hoop_pos + (norm_hoop_pos|participant),
                    data = model_data,
                    family = "beta",
                    prior = HS_prior,
                    cores = 2,
                    chains = 2,
                    iter = HS_iter,
                    warmup = HS_iter/2,
                    control = HS_control)
```

```
##                 Estimate Est.Error    l-95% CI  u-95% CI     Rhat Bulk_ESS
## Intercept     0.15172940 0.1172168 -0.0846409 0.3840119 1.001400     2234
## norm_hoop_pos 0.06977348 0.1484155 -0.2241114 0.3653229 1.002733     2132
##               Tail_ESS
## Intercept         2770
## norm_hoop_pos     2731
```

The model results confirmed that participants in general had a bias towards standing closer to the small hoop (mean of 0.549, 95% HDPI of |0.506, 0.591|). We can be reasonably confident about this results as the $p(x > 0.5|data) = 98.7\%$. This can be seen in the posterior in the right hand plot of Figure 5. Also, note that distance did not appear to have an effect on position (i.e., participants were generally biased slightly towards the smaller hoop).

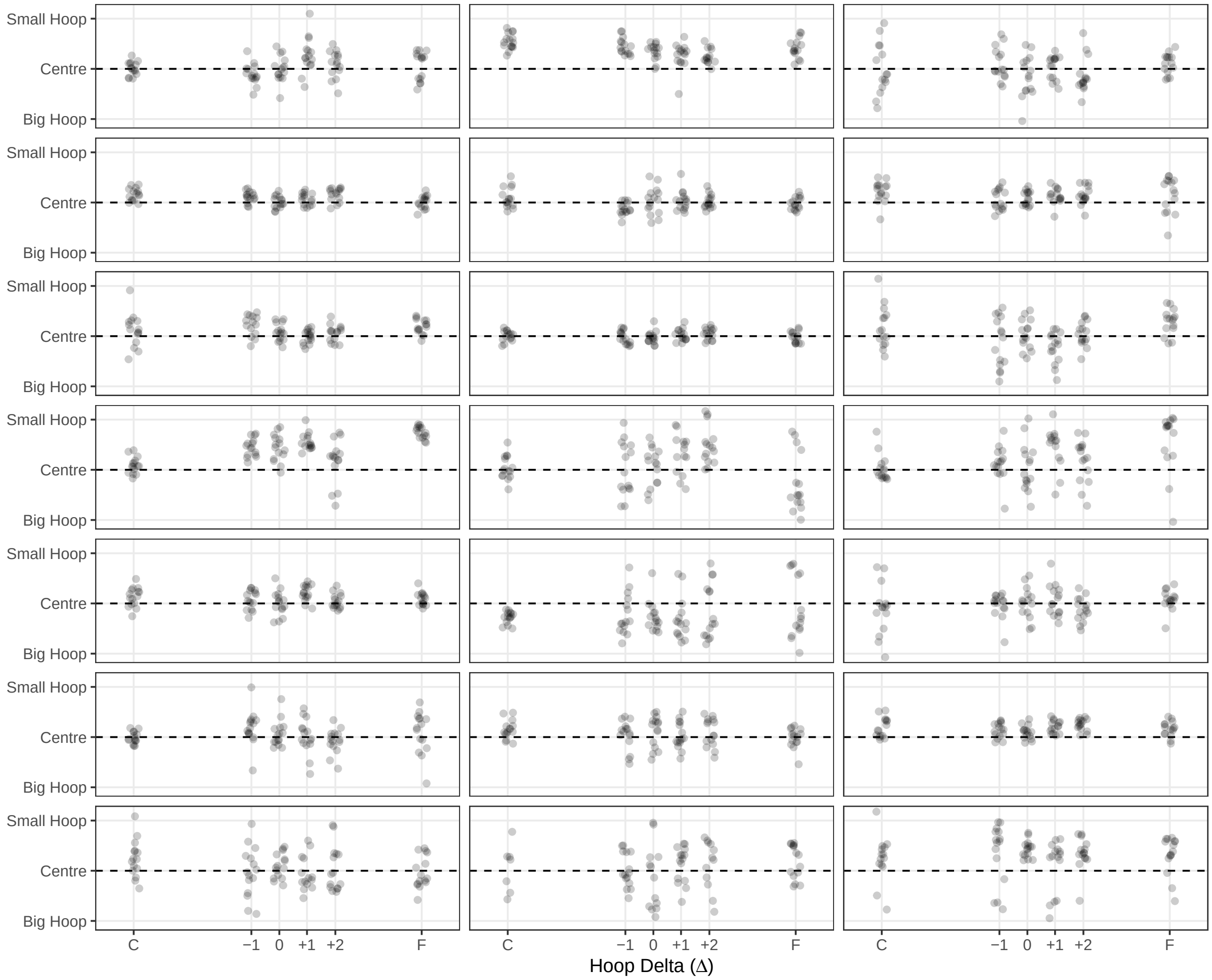

Figure 4: Each facet of this plot shows were each participant stood on each trial. The x-axis shows the different distances that participants were tested at with 0 being the point at which they were closest 50% from the centre. The y-axis shows the normalised standing position between the Big Hoop and the Small Hoop.

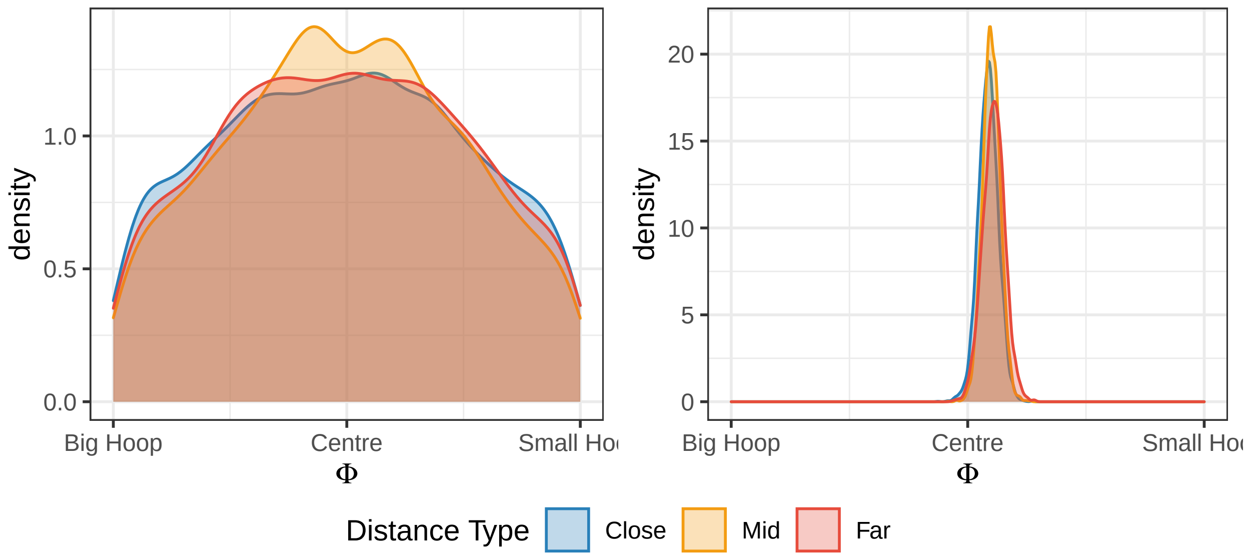

Figure 5: Figure on the left shows the Prior predictions. The figure on the right shows the Posterior predictions once the model had been conditioned on the data

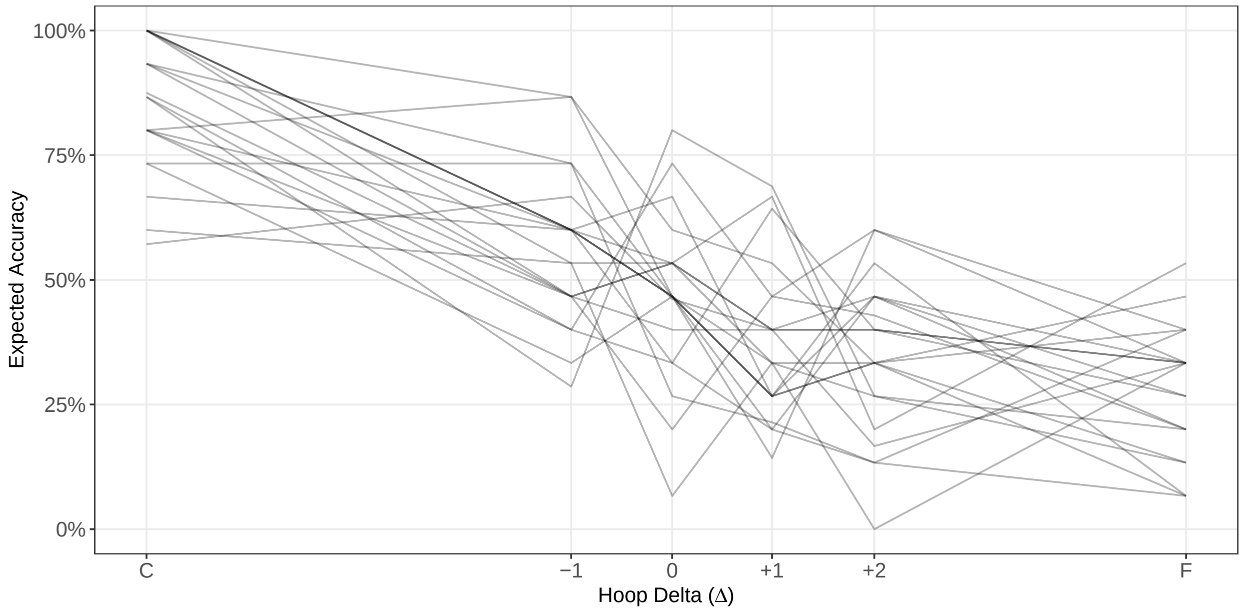

Figure 6: This figure shows the average accuracy participants achieved at each distance. Each line represents a participant

3.1.2 Raw Accuracy

3.2 Experiment 2: Two Throws

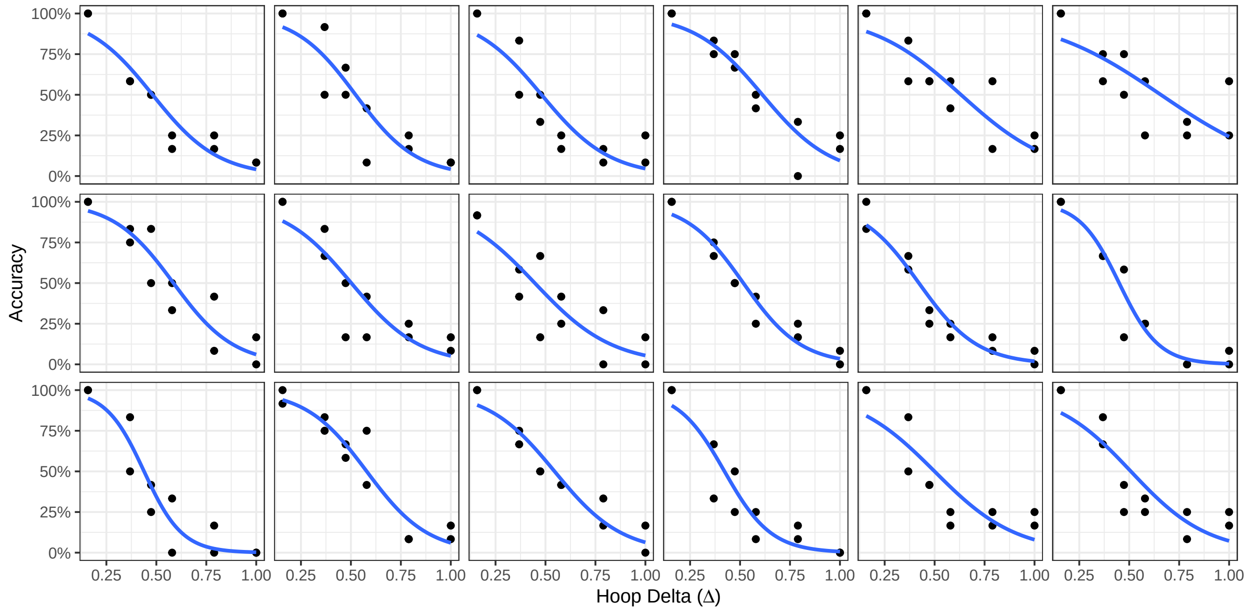

Figure 7: Each facet shows how a participant's accuracy dropped as a function of distance. The x-axis has been normalised so 1 represents the furthest range tested.

Figure 8 shows the individual data for each of the participants in this experiment. Each plot shows where the participants stood on each trial for each of the distances they were tested at with the different conditions being colour coded.

A Bayesian Beta regression was carried out to investigate whether participants performed the task in a more optimal way when they were given the chance to attempt to throw at both targets. The predicted value was the Normalised standing position with 0 being central and 1 being next to one of the hoops. The predictors were the normalised distance of the hoops from the centre (norm_delta), the number of throws participants had (Num_throws), and the interactions between these two terms.

3.2.1 Modelling

We first carry out a prior predictive check to confirm that our choice of priors are reasonable. The model was specified as follows with the prior predictive checks having the additional "sample_prior" argument set to "*only*."

Next, we train the model on the data and plot the posterior distributions.

```
Two_throw_m1 <- brm(
  abspos ~ 0 + norm_delta * Num_throws + (0 + norm_delta * Num_throws|Participant),
  family = "beta",
  data = model_data_pos,
  prior = TT_prior,
  cores = 1,
  chains = 1,
  iter = TT_iter,
```

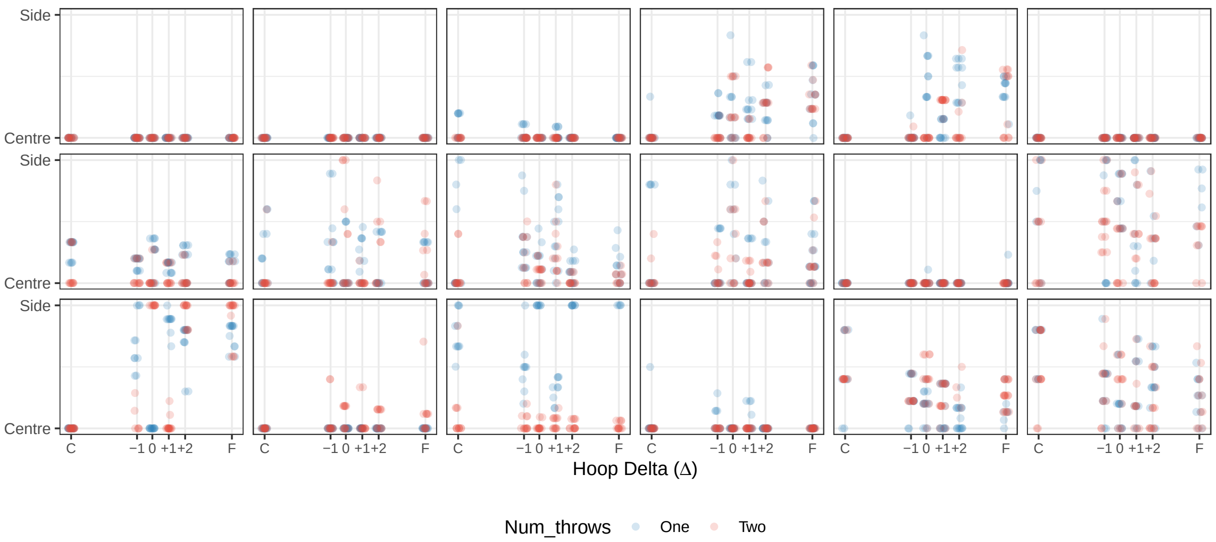

Figure 8: Each facet of this plot shows the stnading position on each trial and condition for each participant. The x-axis shows the different distances that participants were tested at with 0 being the point at which they were closest 50% from the centre. The y-axis shows the normalised standing position (between the centre point and one of the two side hoops).

```
  warmup = TT_iter/2,
  control = TT_control)
```

```
##                              Estimate Est.Error   l-95% CI   u-95% CI      Rhat
## norm_delta                 0.06350622 0.3640363 -0.6734496  0.7700871 0.9999664
## Num_throwsOne             -1.32335625 0.3361194 -1.9473065 -0.6387760 1.0020476
## Num_throwsTwo             -1.94651783 0.2722204 -2.4720970 -1.3892078 1.0015840
## norm_delta:Num_throwsTwo   0.29189537 0.2576929 -0.2187942  0.7945267 1.0002590
##                           Bulk_ESS Tail_ESS
## norm_delta                    2210     3057
## Num_throwsOne                 1547     2388
## Num_throwsTwo                 2028     2618
## norm_delta:Num_throwsTwo      7074     4036
```

As can be seen from the summary output above, $\hat{R} \approx 1$.

The analysis suggested that there was an overall greater tendency for participants in the One-throw condition to stand further from the centre (mean of 0.222, 95% HPDI of |0.123 , 0.33|) than when they were in the Two-throw condition (mean of 0.152, 95% HPDI of |0.082 , 0.228|) with P(One-throw > Two-throw|data) = 91%.

This effect was the strongest for the closest separation which reflects the larger amount of variation in standing position with distance in the Two-throw condition (Figure 9. In general, when in the One-throw condition, participants stood further from the centre (mean of 0.218, 95% HPDI of |0.123 , 0.314|) than in the the Two-throw condition (mean of 0.218, 95% HPDI of |0.123 , 0.314|) with P(One-throw > Two_throw|data) = 97.62%. However, as can be seen in 9, the difference is generally small which and consistent across all distances. This means that when participants were given the opportunity to throw to both hoops (i.e., in the Two throw condition), they were still sub-optimal in their performance.

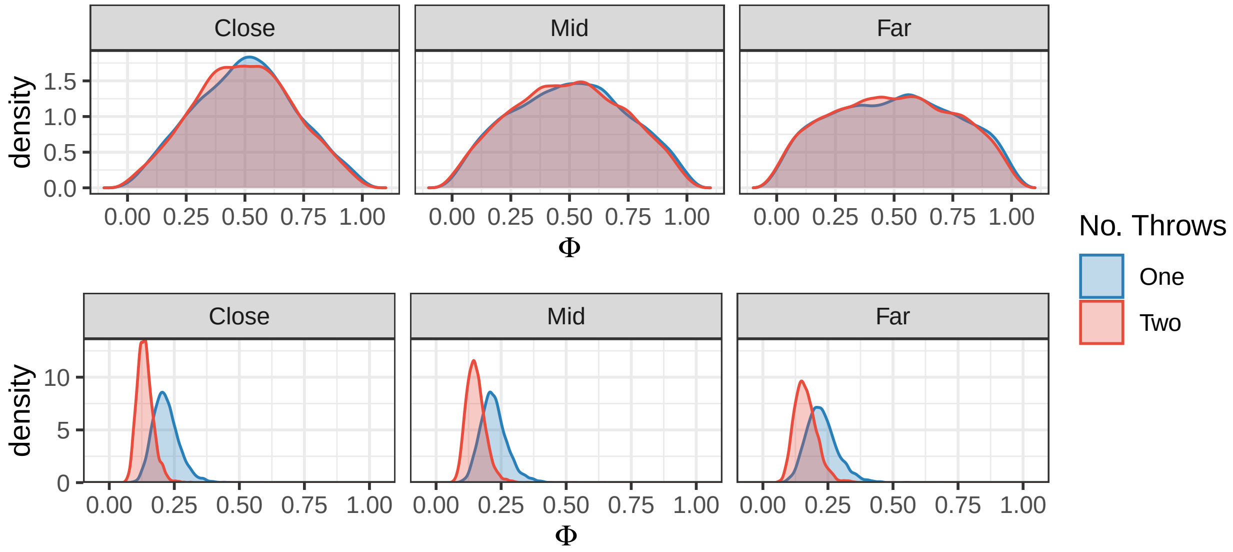

Figure 9: The top panel shows the Prior preditcions for the Two Throw experiment. The bottom panel shows the Posterior predictions for the Two Throw expeirment.

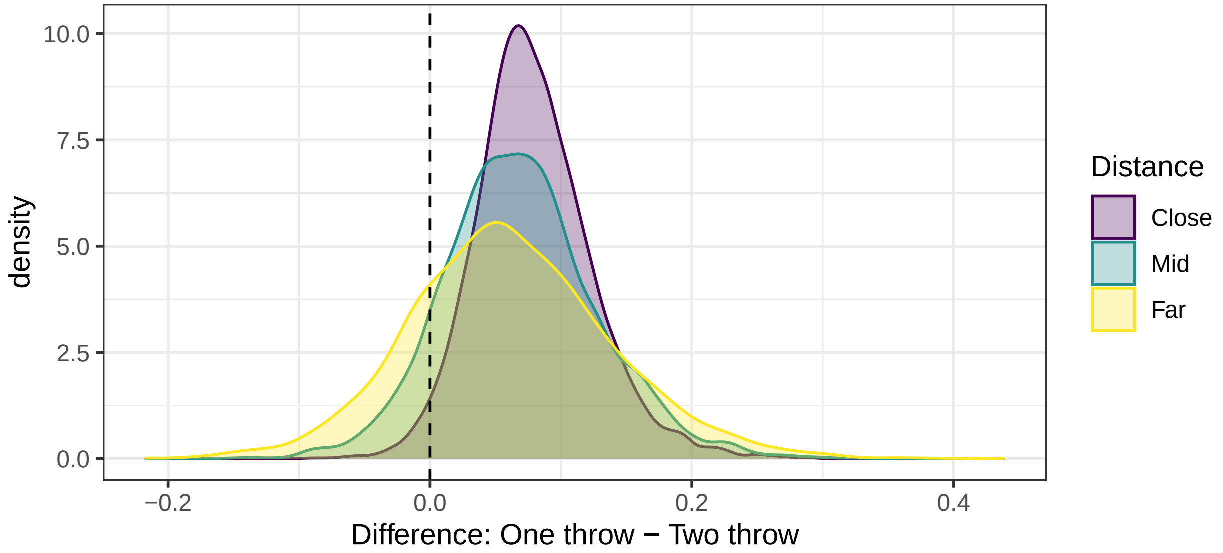

Figure 10: This figure shows the difference in the estimates for the Two Throw experiment. This is the diffrence between the red and blue distributions see in the Figure '9' with a bigger number representing the One Throw condition have a larger mean than the Two Throw condition.

3.2.2 Raw Accuracy

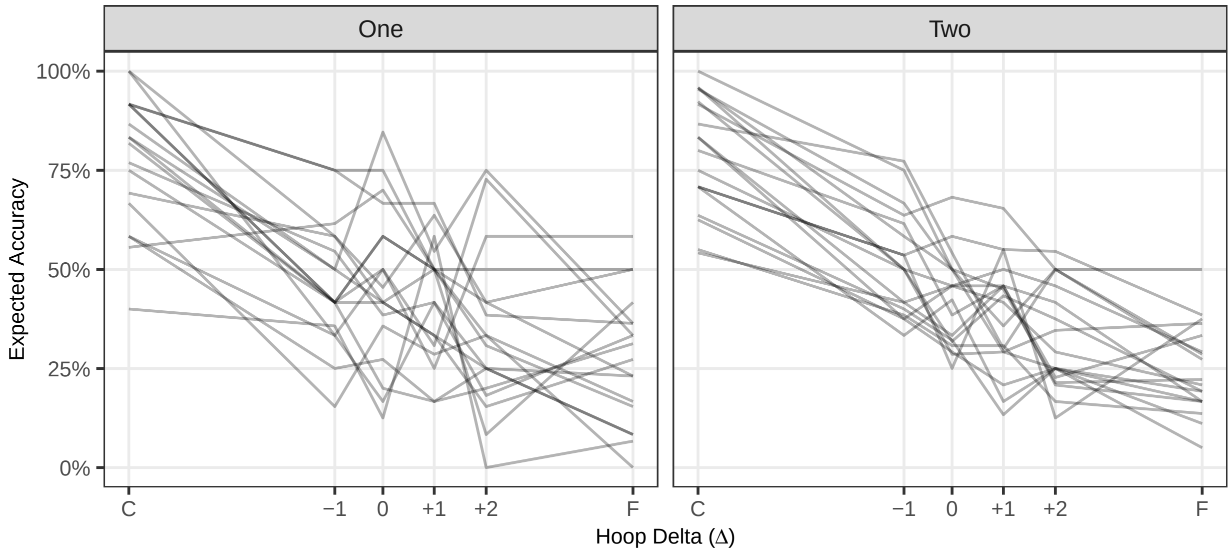

Figure 11: This figure shows the average accuracy participants achieved at each distance. Each line represents a participant The facets represent the two different conditions.

3.3 Experiment 3: Reward

```
## ‘geom_smooth()‘ using formula ’y ~ x’
```

Figure 13 shows the individual data for each of the participants in this experiment. Each plot shows where the participants stood on each trial for each of the distances at which they were tested with the different conditions being colour coded.

A Bayesian Beta Regression was carried out in order to investigate whether opting for an unequal reward structure in this task would facilitate the use of a more optimal strategy in the *Throwing Task*. The predictors in this model were whether the participant had opted for an *Equal* or *Unequal* (Gamble_Type), whether the hoops were *Close* or *Far* (dist_type), and the interaction between these. Additionally, random effects of participant were included for all predictors. The predicted value was the normalised standing position, with 0 representing the centre and 1 being next to one of the hoops.

3.3.1 Modelling

The priors in for this model were set so as to reduce the likelihood of extreme values (i.e., close to 0% or 100%). Until this experiment, we had not run a version of this task in which the different targets could have different value associated with them.

As the prior predictive checks demonstrated that the model was able to retrieve the prior, and the priors were suitably flat, the model was conditioned on the raw data from the experiment. The model looked as follows with the prior predictive checks having the additional "sample_prior" argument set to "*only*":

```
# run model
Reward_m1 <- brm(
  Norm_Dist ~ 0 + close_equal + close_unequal + far_equal + far_unequal +
```

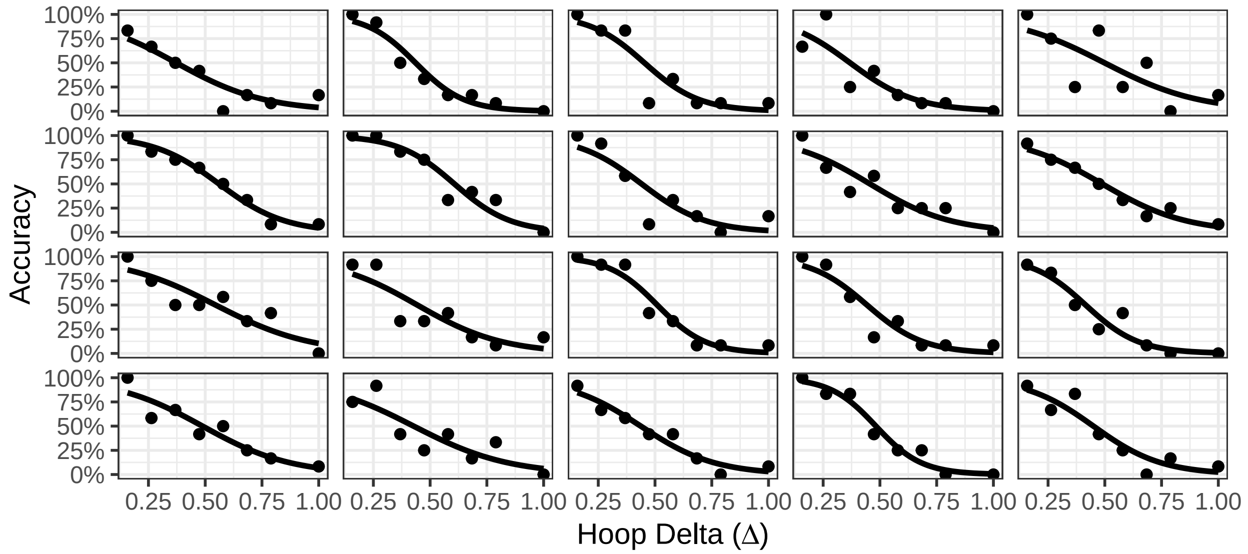

Figure 12: Each facet shows how accuracy changed over distance for each pariticipant. The x-axis has been normalised with 1 being the furthest

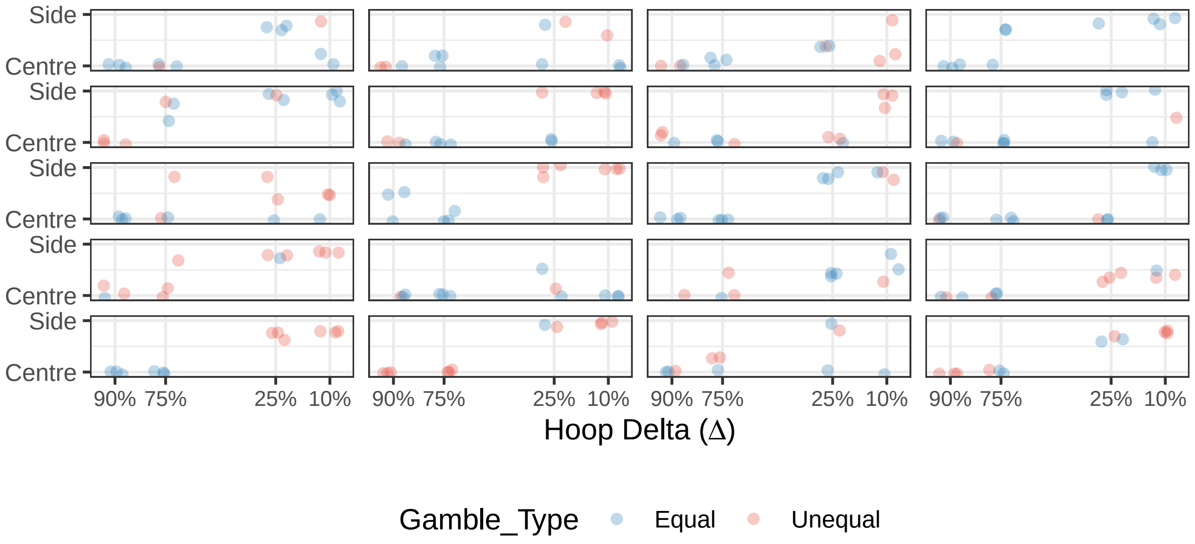

Figure 13: Each facet in this plot shows the decision on a single trial about where to stand and how the participant had opted to split the reward. The x-axis corresponds to the participants chance of success had they stood at the centre with the y-axis showing the normalised standing potision.

```
    (0 + close_equal + close_unequal + far_equal + far_unequal|Participant),
  data = model_data,
  family = "beta",
  prior = R_prior,
  chains = 1,
  iter = R_iter,
  warmup = R_iter/2,
  control = R_control)
```

```
##                   Estimate Est.Error     l-95% CI   u-95% CI      Rhat Bulk_ESS
## close_equal   -2.0308981 0.1631362 -2.35258856 -1.7193381 0.9998354     4217
## close_unequal -1.9542541 0.2095497 -2.37167467 -1.5483326 0.9999228     5317
## far_equal     -0.3462181 0.3463279 -1.03788578  0.3409172 1.0005822     3355
## far_unequal    0.4749207 0.2685055 -0.07116689  1.0080401 0.9999710     4151
##               Tail_ESS
## close_equal       3931
## close_unequal     4097
## far_equal         3220
## far_unequal       3960
```

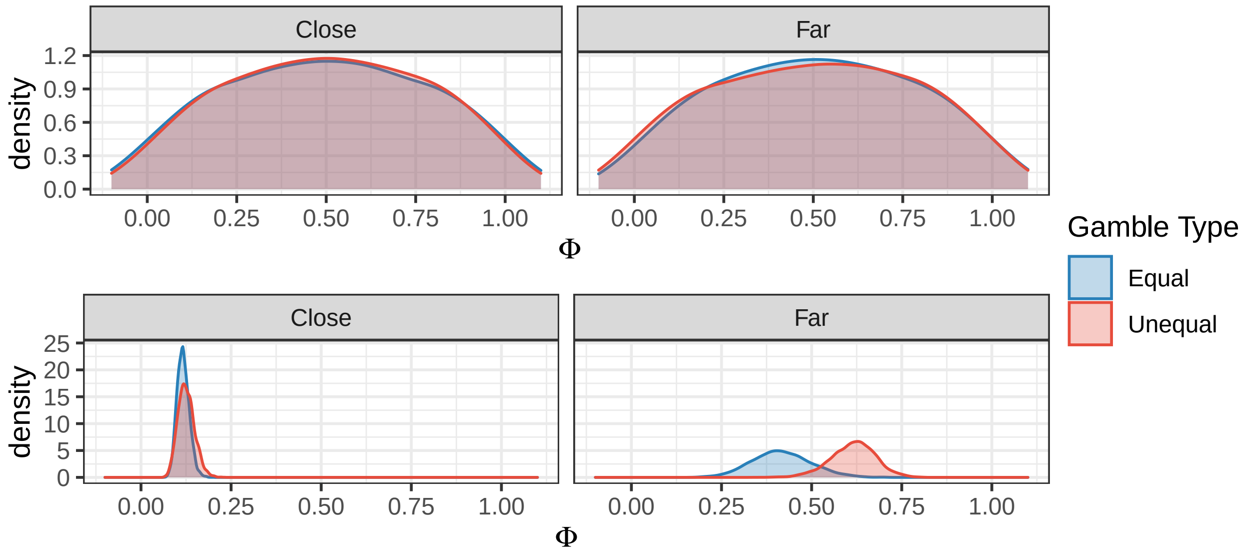

Figure 14: This plot shows the posterior distribution of standing positions for the Reward study

As can be seen in Figure 14, participants in this task were more likely to stand towards one of the side hoops when the hoops were far apart (mean of 0.516, 95% HPDI of |0.287 , 0.711|) than when they were close together (mean of 0.121, 95% HPDI of |0.084 , 0.164|) with P(Far > Close|data) = 100%. This suggests that participants were sensitive to the addition of a reward in this experiment.

In addition to this, the interaction of Distance Type with Gamble Type was in the direction of an asymmetrical reward structure pushing participants to make more optimal decisions with participants standing closer to one of the targets when they opted for an unequal split (mean of 0.615, 95% HPDI of |0.491 , 0.74|) than when they had opted for an equal split (mean of 0.417, 95% HPDI of |0.255 , 0.576|) with P(Unequal > Equal|data) = 96.6%.

3.3.2 Raw Accuracy

```
## 'summarise()' has grouped output by 'Participant', 'slab_measures'. You can override using the '.grou
```

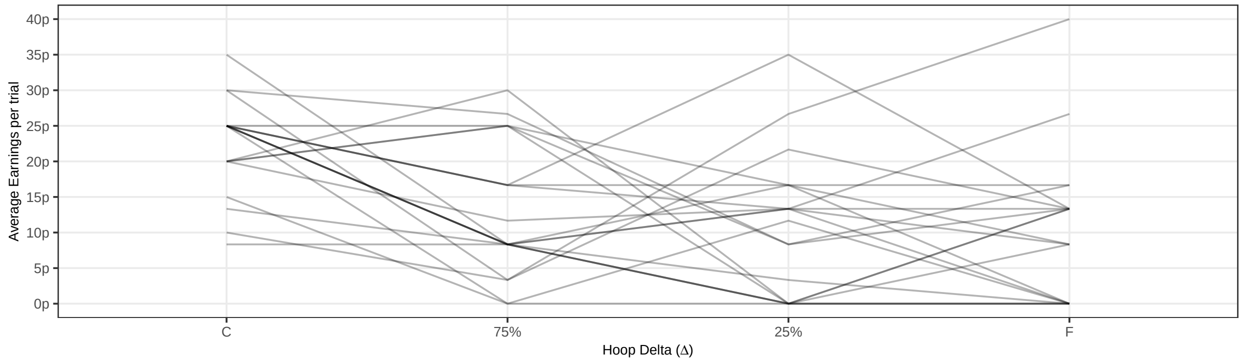

Figure 15: This figure shows the average earnings participants achieved at each distance. Each line represents a participant The facets represent the two different conditions.

4 Probability Matching Experiment

4.1 Note

This experiment was cut from the main body of the paper as it is the only experiment to make use of the *Detection Task* from Clarke and Hunt (2016). The logic behind this experiment was the same as in the experiments discussed in the main paper in that we investigated a potential asymmetry in the design that may alter the way in which participants approached this task.

4.2 Introduction

In this experiment, we made the probability of the target appearing in one of the two locations higher (80%) than the other (20%). When predicting which of two events is about to occur (for instance, whether a blue or yellow light would illuminate on a particular trial), people tend to match the underlying probability of each event, a tendency called Probability Matching (Koehler and James 2010; Goodnow 1955; Vulkan 2000). If event A occurs 80% of the time, and option B only 20% of the time, the best course of action is to predict event A every time, as this would result in an average accuracy rate of 80%. People, in general, tend to instead select each option in proportion to its likelihood of success, yielding an average success rate (in this example) of only 68%.

Probability matching may be due in part to a misunderstanding of probability (Reimers, Donkin, and Pelley 2018), but it has also been argued to reflect a reasonable tendency to seek out and exploit patterns in sequential events (Gaissmaier and Schooler 2008; Wolford et al. 2004). Gao and Corter (2015) argue that when a person selects the more likely option on every trial (a strategy known as *Maximising*), they are accepting a loss for a minority of trials (20% in this example). As demonstrated in Kahneman and Tversky (1984), people tend to find certain loss very aversive, so a strategy that includes a certain loss may be disregarded as a "solution" to the problem at hand (Goodnow 1955). The only way to avoid this loss is to make an attempt to detect and exploit any potential pattern. In the context of deciding where to fixate between two potential targets locations, in both Morvan and Maloney (2012) and Clarke and Hunt (2016), the probability for each location to contain the target target was 50%. Given the philosophical and experimental arguments described above, participants may react to this choice between equally likely

options with idiosyncratic pattern-seeking behavior (Gaissmaier and Schooler 2008; Yellott 1969; Wolford et al. 2004). In Clarke and Hunt (2016), this may have interfered with participants using information about their own ability to make decisions. Instead, they may have been attempting to figure out patterns in the sequence of targets to make better guesses about which one was likely to be the target on each trial. Adding in a bias for one option to be more likely may cater to people's tendency to seek out, and attempt to exploit, patterns. It also breaks the Buridan's Ass deadlock and makes the decision about which of two goals to attempt easier. In this Experiment, unequal probability was introduced into the paradigm used by Clarke and Hunt (2016) in order to investigate 1) whether participants would make use of probability information in deciding where to fixate, and 2) whether probability manipulations might facilitate the decision about whether to fixate between two potential target locations or to look directly at one of them. If so, it would suggest that at least part of the reason for the poor decisions observed previously was the (unnatural) balance between the two potential targets. Additionally,this experiment will provide a useful replication of both the fixations decision results (Morvan and Maloney 2012; Clarke and Hunt 2016) and the probability matching tendency (Vulkan 2000) in a new context.

4.3 Methods

4.3.1 Power Analysis

For this experiment, data from ($N = 12$) Clarke and Hunt (2016), which acted as a substitute for the Symmetric condition, and an unpublished pilot study ($N = 11$), which followed the same rules as the Bias condition in the main experiment, were resampled in order to investigate how often participants would fixate one of the side boxes given it was more likely to contain the target for the Bias condition. For the Symmetric condition, we followed the same rule as the in the paper and classified the most likely box as the side box that participants fixated most often (Figure 16. The main interest for our purposes was the proportion of time participants fixated the most likely box.

```
## `summarise()` has grouped output by 'Participant', 'Condition'. You can override using the `.groups`
```

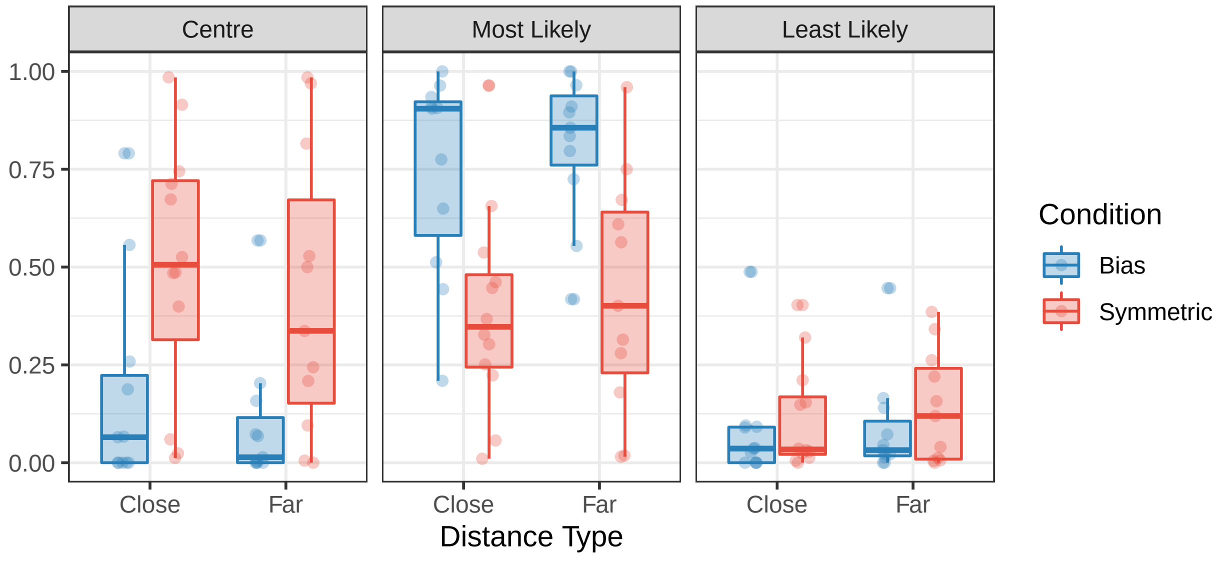

Figure 16: Boxplots to show the proportion of trials in which the participants fixated the each box by Condition and Distance type

The data were coded so that a fixation was classified as either being to the Most likely box or not. We then resampled (with replacement) these data by selecting a random N participants from each condition

(ranging from 2 to 20) then sampling 300 trials from these participants from the different data sets. This was done 5000 times to estimate the expected difference between the Symmetric and Bias conditions in terms of proportion of fixations to the Most likely side and the associated certainty around these values.

As can be seen in the figure below, the uncertainty surrounding the estimate for the difference between the groups appears to plateau somewhere around 15 participants. The shaded region represents a 95% Highest Density Interval (HDI) for the distribution of differences simulated through resampling. As such, the sample size of 18 in the main experiment appears to be ample in order to detect this difference and increasing the sample size above this value does not add more to the certainty, as can be seen in Figure 17.

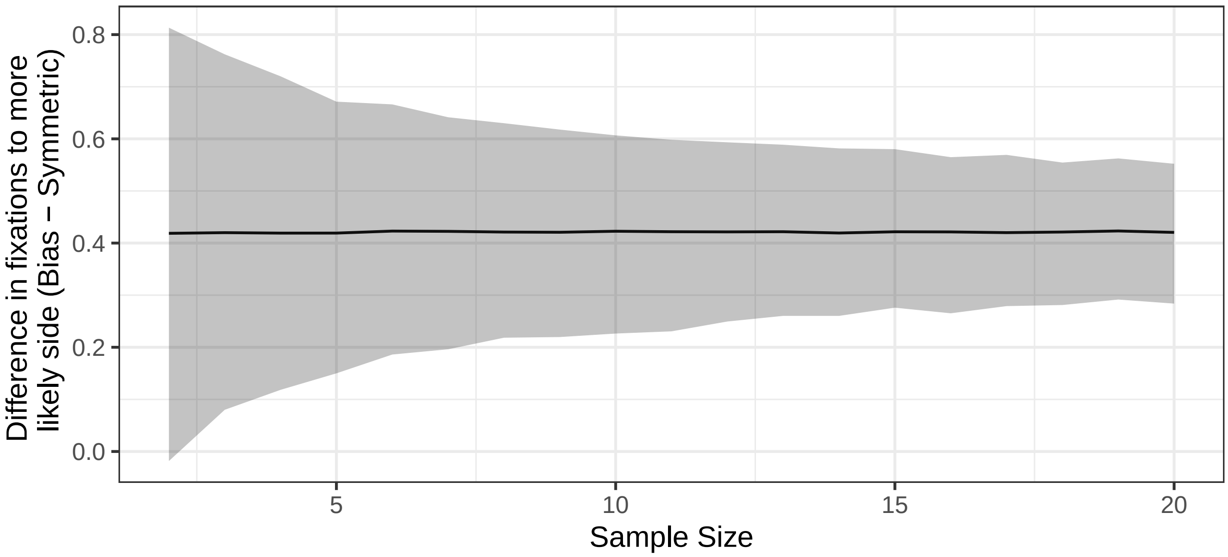

Figure 17: Plot to show how the 95% HDI of the estimated difference in fixations to the most likely box changed with an increase in participants

4.3.2 Participants

16 Participants (two Male) were recruited from the University of Aberdeen community with an average age of 22.75 (between 19 and 29). Each participant was reimbursed £10 for their time.

4.3.3 Procedure

The experiment followed a similar procedure to the "*Detection Task*" from Clarke and Hunt (2016). In the first session, we measured visual acuity which lasted approximately 30 minutes. This was followed by a second session in which the participants performed the actual decision task which lasted approximately 40-50 minutes.

The experiment took place in a darkened room on a desktop computer. An Eyelink 1000 (version 4.594)(SR Research ltd, Mississauga, Ontario, Canada) was used to record eye position at 1000Hz. In each session, a 5 point calibration was carried out with additional calibration and validation sequences prior to each block, and if the participants had broken fixation ten times cumulatively or for five trials in a row. The stimuli were displayed on a CRT monitor (resolution 1920 X 1080 pixels) using Matlab 7.9.0 (R2009b) with Psychtoolbox (Brainard 1997; Pelli 1997) and EyelinkToolbox functions (Cornelissen, Peters, and Palmer 2002). A chin rest with forehead bar was used to ensure participants maintained a viewing distance of $\approx$ 47cm.

In the first session, participants were instructed to remain fixated at the center and identify a letter that would appear in one of two boxes. At the start of each trial,participants fixated a central black cross on a

grayscale background and pressed the spacebar. The cross was flanked by two boxes which were a lighter shade of grey and occupied 1 ◦ of visual angle. After a stable fixation had been maintained for 700ms, the target would appear in one of the two boxes for 500ms. The target was a white letter, 0.4° of visual angle, drawn using the Sloan font as these letters are generally of equal recognisability at different viewing angles(Sloan, Rowland, and Altman 1952). Ten letters were used and one was selected randomly on each trial to be the target letter. Participants were then presented with a screen prompting them to report which letter was presented by clicking on the corresponding character. An illustration of each trial in Session 1 can be seen in Figure **??**.

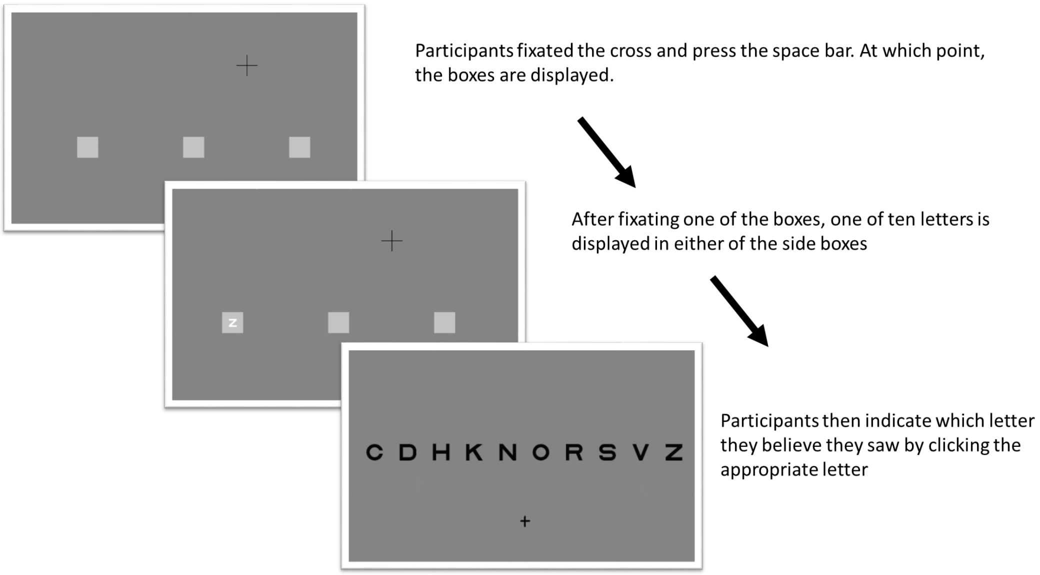

Figure 18: The sequence for every trial in Session 1

The boxes were presented on either side of the fixation cross at several different eccentricities (3.1°,4.3°,5.8°,7.5°,9.3°,11.1°,12.5°,& 13.7°). Each eccentricity was repeated 12 times in a row before moving on to another eccentricity (the order of these sets of 12 eccentricities was random),for a total of 96 trials in a block.

After each block, participants were offered a break before recalibrating and moving onto the next block. Participants completed four of these blocks. The data from this first session were used to tailor the separations that would be used for the second session. A "switch-point" was calculated for each participant based on their Session 1 performance. This was then used as an anchor point from which to calculate the other separations to be used in Session 2. The switch-point for each participant was the separation at which the participant was 68.5% accurate. The accuracy level of 68.5% is the mid-point between 55% and 82%; these two values are the points at which participants should switch from fixating the central box to the side box in the Symmetric and Bias conditions, respectively(see below for more details).

From this point, 6 other separations were calculated ± 1°, 2°, and 3°. There were two other points included which acted as anchors, one being a very large distance (19.4°), the other very small (1.9°). In Session 2, participants started by fixating a cross that appeared above where the boxes would appear. The cross would appear at the midpoint between the centre box and either the left or right box with an equal probability. Once they had fixated the cross, they were instructed to press the spacebar. After a stable fixation was detected for 700ms, three boxes would be presented (Figure 19). One box would appear in the centre with the other two spaced equally on either side with separations calculated as above, based on Session 1 performance for that particular participant. Once these boxes had appeared, participants were instructed to fixate one of the three boxes. Participants were told that the target would never appear in the central box, however they could choose to fixate this location. After they had fixated one of the boxes, the letter appeared in one of the side boxes for 500ms. After this, the 10 letter stimuli were presented on screen for

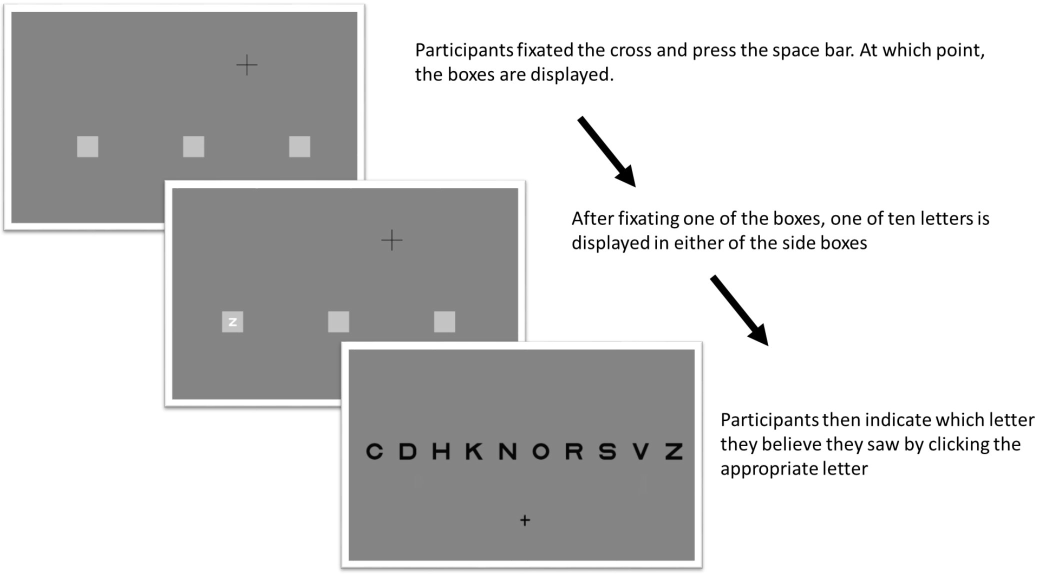

Figure 19: The sequence for every trial in Session 2

them to select which letter they had seen. Each separation appeared 10 times within a block, for a total of 90 trials. The order of separations was randomised (rather than blocked, as it was in Session 1).

Prior to each block, participants were told how likely each box was to contain the target. There were two levels of probability; one in which each box was equally likely to contain the target on each trial (Symmetric), and one in which one side would contain the target 80% of the time (Biased). Each participant took part in both the Symmetric and Biased conditions. Participants completed one condition for 4 blocks before moving on to the other condition. This order was counterbalanced across participants. Additionally, the side that was more likely to contain the target was also counterbalanced, to control for any bias to look to one side over the other.

4.3.4 Optimal Strategy

The optimal strategy is to fixate whichever of the three boxes maximises expected accuracy. We refer to a participant's expected accuracy if they had followed the optimal strategy, as "optimal accuracy." Using the psychometric curves fit to each participant's Session 1 data, we can calculate how likely a given person is to detect the target at various distances. This was then used to predict how accurate that same person would be if they had fixated one of the side boxes, or the centre box, by using their average accuracy for a given distance from the left and right box (Bl and Br respectively) in Session 1. The fixation choice that gave the greatest expected accuracy was then selected as the optimal decision for the respective box separation. As probability was also a factor in this experiment, this had to be accounted for by multiplying the chance of detecting the target in either box by the probability that the box would contain the target (Pl and Pr). The formula for expected accuracy is therefore: $(Bl \times Pl) + (Br \times Pr)$. Expected accuracy given optimal fixations differs between the Symmetric and Biased conditions. For example, in the Symmetric condition, participants could expect a 55% success rate if they fixated the optimal location when the targets were far apart. This value comes from assuming they would be $\approx$ 100% accurate for the fixated box, and at chance level for the non-fixated box (10%). This gives us $(1 \times 0.5) + (0.1 \times 0.5) = 55$. To get the lower limit for the Bias condition, we simply change the chance for each box to contain the target (additionally, we assume that the participant would fixate the most likely box). This gives us $(1 \times 0.8) + (0.1 \times 0.2) = 82$. This same formula can be used to calculate expected accuracy had the participant fixated the central box. In this case, $Bl = Br$, and so would simply be put into the formula using the appropriate values for P, as demonstrated above.

4.3.5 Analysis

All analysis for this experiment follow the same procedures as described in previous sections.

4.4 Results

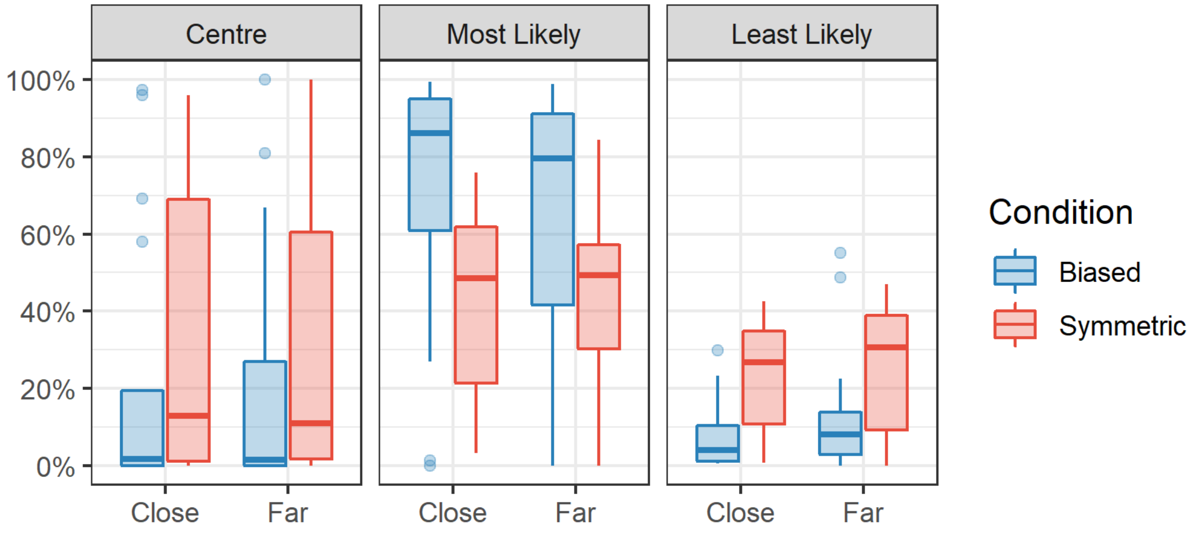

Figure 20: These boxplots show the proportion of the time that participants fixated the centre (left panel), most-likely (central panel) and least-likely (right panel) box. In the Bias condition, most likely was the box with and 80% probability of containing the target. In the Symmetric condition, most-likely was whichever side box a given participant had fixated the most. The Close and Far distinction is baseed on when participants should swith from fixating the central box to the side box. Note that for some participants, expected performance differences between the Centre and Side strategies for the closest separation was negligible. However, all participants should have fixated the Side boxes in the Far condition, where the performance advantage of doing so was substantial.

The choices made by participants on where to look are summarized in Figure 20. The optimal strategy in this situation is to fixate the central box when the two sides boxes are near (i.e., when their distance from centre, Δ, is closer than each participant's switch point). When they are far (i.e. delta is greater than the switch point), a participant behaving optimally should fixate either of the side boxes when in the symmetric condition, and the most-likely box when in the biased condition. The first panel of Figure 20 clearly shows that our participants did not follow this strategy. However, while adding a bias to the location of the target did not help participants to behave optimally, we can see that it did have an effect on their behavior, as they were much less likely to fixate the central box. Furthermore, when fixating one of the two side squares, they fixated the square that was most likely to contain the target (Figure 20, central and right panel) almost all the time

We can also look at how the choice of where to look influenced participants' accuracy. We use the target detection models from Session 1 to calculate the expected accuracy given either (i) an optimal, (ii) counter-optimal, or (iii) the observed strategy. This measure eliminates the variance present in the actual accuracy data due to the random location of the target and participant's guessing the correct answer by chance. These data are summarised in Figure 21. We can see that in the symmetric condition, with the exception of one individual, our participants behaved in a way that gave rise to expected accuracies much closer to the counter-optimal strategy than optimal. Expected accuracy was higher in the biased condition, with four participants reaching optimal performance, with the rest ending up somewhere between the optimal and counter-optimal.

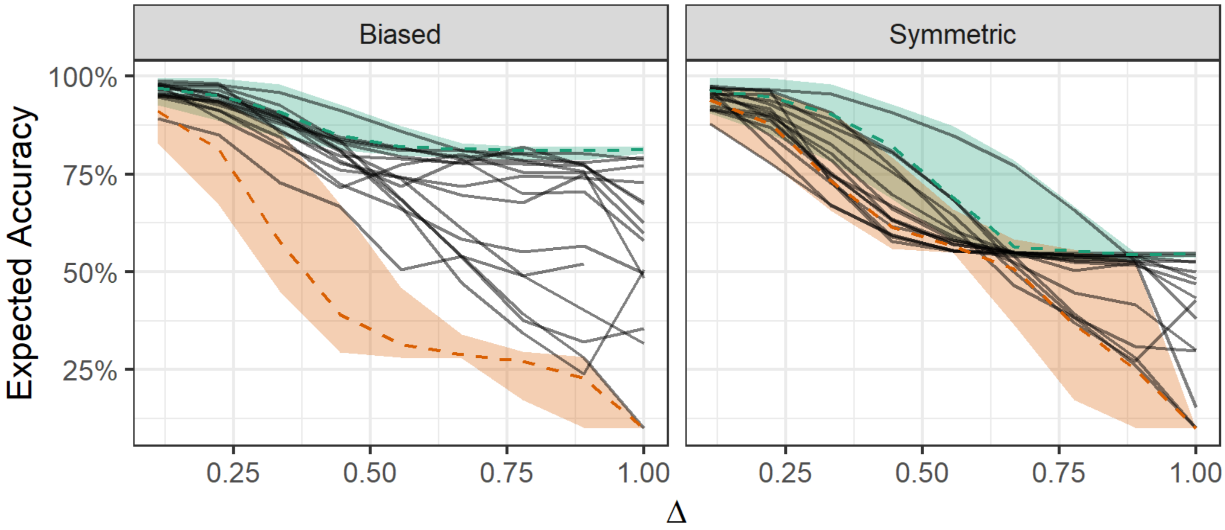

Figure 21: These show the expected accuracy for each participant (black lines) plotted against Δ. The different shaded regions show the minimum (orange) and maximum (green) expected accuracy rates for all participants. Δ is the separations at which each participant was tested rescaled to be between 0 and 1

4.4.1 Session 1 Accuracy

4.4.2 Modelling

```
ProbModel <- brm(Ml_fix ~ (bias_type + dist_type)^2 + (dist_type * bias_type|participant),
                 data = df_model,
                 family = "bernoulli",
                 prior = Rewards_priors,
                 chains = 1,
                 iter = n_iter,
                 warmup = n_warmup)
```

4.4.2.1 Prior Predictive checks

```
##                                  Estimate Est.Error   l-95% CI   u-95% CI
## Intercept                      0.50020792 0.6149599 -0.7125894 1.56002142
## bias_typeSymmetric            -0.94610142 0.4938304 -1.8217266 0.02537652
## dist_typeFar                  -0.29545654 0.2399881 -0.7525764 0.19960728
## bias_typeSymmetric:dist_typeFar  0.06502651 0.2649023 -0.5061199 0.53576669
##                                    Rhat Bulk_ESS Tail_ESS
## Intercept                      1.001484      231      413
## bias_typeSymmetric             1.000067      290      549
## dist_typeFar                   1.003212      560      384
## bias_typeSymmetric:dist_typeFar 1.001385      667      618
```

The results of this model suggest that our participants were sensitive to the probability information (see figure @ref{fig:ProbModelOutputs}). In the Biased condition, the average participant fixated the most likely target 58.1% of the time (95% HDPI of |31.3%, 82.2%|), compared to 36.9% (95% HPDI of |17.5%,54.7%|)

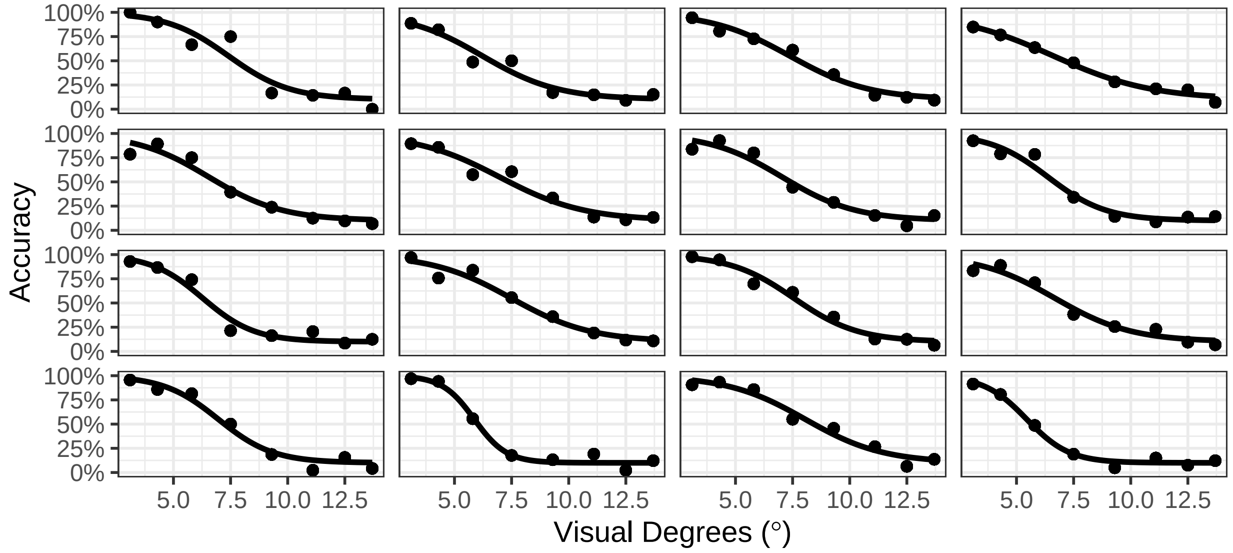

Figure 22: This figure shows the rate at which participants correctly identified the target letter presented within a box placed some distance from the centre with the lines showing a logistic regression on these data.

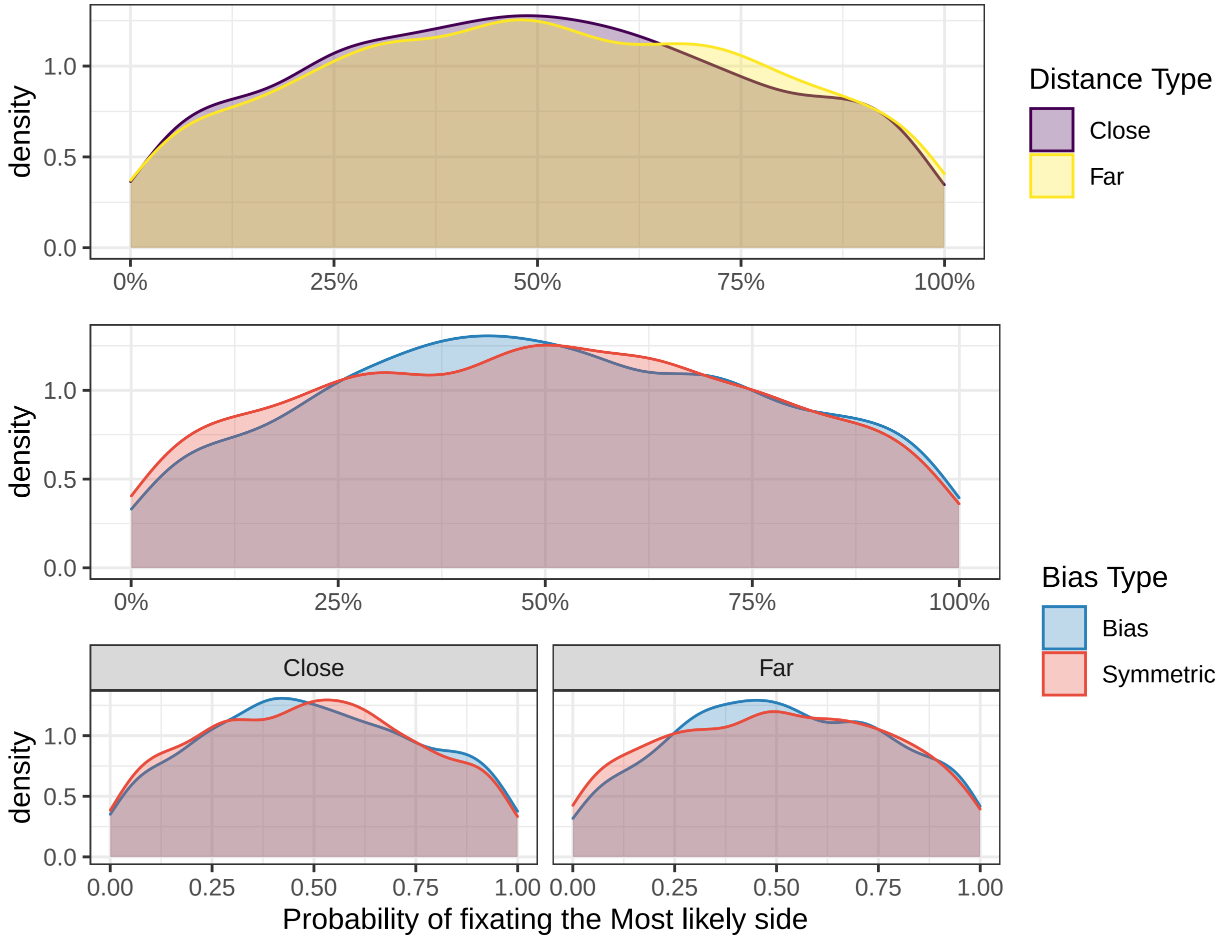

Figure 23: Prior Predictive checks for the Probability matching experiment

in the symmetric condition. The width of these intervals reflects a high degree of uncertainty in fixed effects, due to the range of behaviours exhibited by participants. None-the-less, the HPDI on the difference between these two conditions, |-0.7%, 41.4%| is largely positive and we can be reasonably confident (P(difference > 0 | data) = 95.9%) that the most-likely target is fixated more frequently in the biased condition. The distance between the square targets did not appear to have any consistent effect in the Symmetric condition, however there was a small decrease in fixations towards the "most likely" box when the boxes were far apart in the bias condition (dropping from 61.4%, 95% HPDI of |34.4%,83.6%| to 54.8%, 95% HPDI of |0.305%,80.3%|). As such, the difference between the conditions were much more pronounced in the close condition (P(Bias > Symmetric|data) = 96.9%) than in the far condition (P(Bias > Symmetric|data) = 94.8%). The width of these intervals reflects the range of performance that was exhibited by participants. However, this does show that participants generally made us of this probability information in order to decide where to fixate.

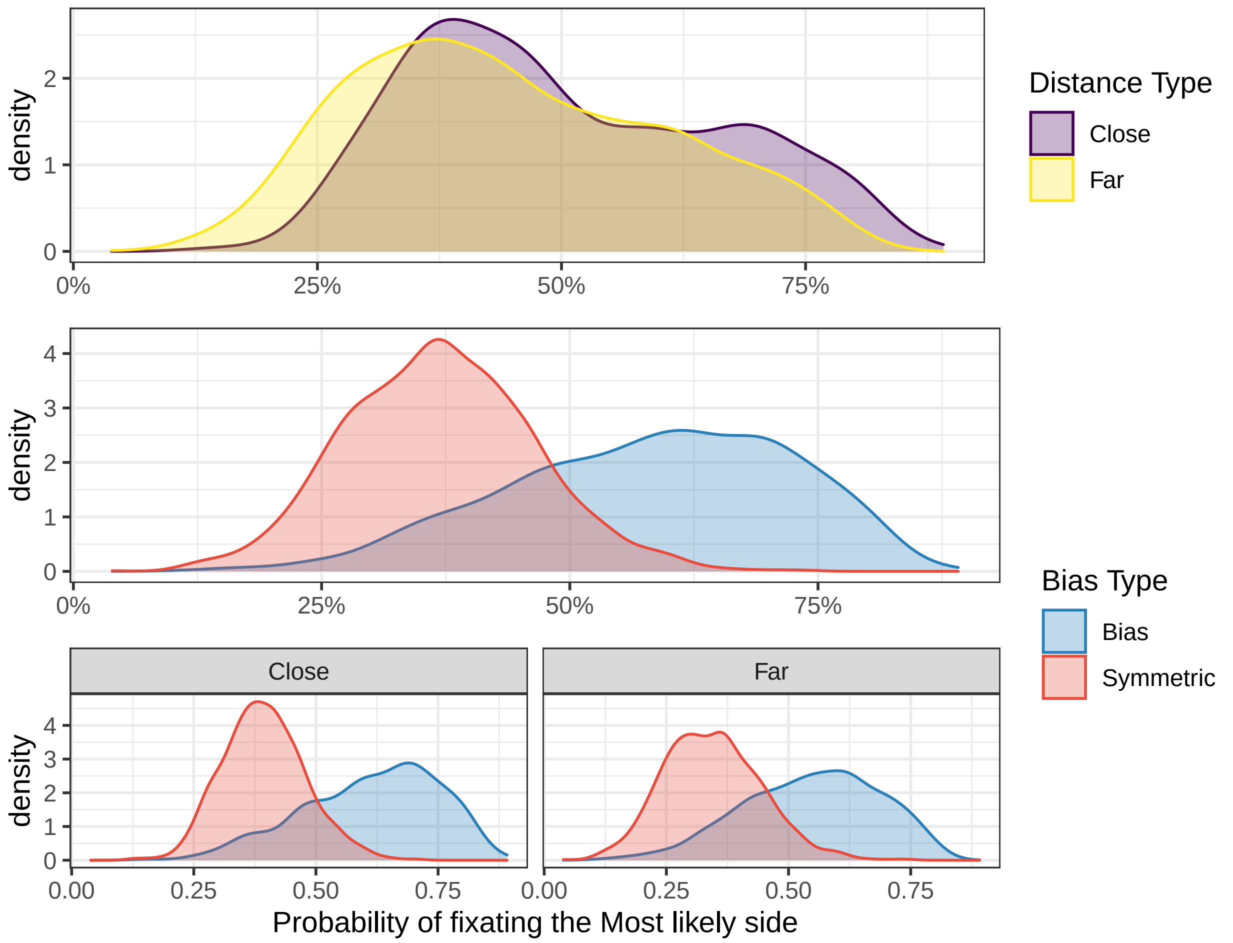

Figure 24: Posterior predictions for the probability matching experiment

4.5 Discussion

The results of this study suggest that participants were in fact sensitive to the difference in probability of each side containing the target and responded by fixating this location more often when there was a bias compared to the Symmetric conditions. However, the presence of a bias did not lead participants to utilising a more optimal strategy. It would appear that the introduction of a bias for one target distracted participants from discovering the optimal strategy. Instead, participants appeared to focus primarily on the probability aspect of the task.

Bibliography

Brainard, David H. 1997. "The Psychophysics Toolbox." *Spatial Vision* 10 (4): 433–36.

Clarke, Alasdair D. F., and Amelia R. Hunt. 2016. "Failure of Intuition When Choosing Whether to Invest in a Single Goal or Split Resources Between Two Goals." *Psychological Science* 27 (1): 64–74. https://doi.org/10.1177/0956797615611933.

Cornelissen, Frans W, Enno M Peters, and John Palmer. 2002. "The Eyelink Toolbox: Eye Tracking with MATLAB and the Psychophysics Toolbox." *Behavior Research Methods, Instruments, & Computers* 34 (4): 613–17.

Gaissmaier, Wolfgang, and Lael J Schooler. 2008. "The Smart Potential Behind Probability Matching." *Cognition* 109 (3): 416–22.

Gao, Jie, and James E. Corter. 2015. "Striving for Perfection and Falling Short: The Influence of Goals on Probability Matching." *Memory & Cognition* 43 (5): 748–59.

Goodnow, Jacqueline Jarrett. 1955. "Determinants of Choice-Distribution in Two-Choice Situations." *The American Journal of Psychology* 68 (1): 106–16. http://www.jstor.org/stable/1418393.

Kahneman, Daniel, and Amos Tversky. 1984. "Choices, Values, and Frames." *American Psychologist* 39 (4): 341–50.

Koehler, Derek, and Greta James. 2010. "Probability Matching and Strategy Availability." *Memory & Cognition* 38 (6): 667–76.

Morvan, Camille, and Laurence T Maloney. 2012. "Human Visual Search Does Not Maximize the Post-Saccadic Probability of Identifying Targets." *PLoS Comput Biol* 8 (2): e1002342.

Pelli, Denis G. 1997. "The VideoToolbox Software for Visual Psychophysics: Transforming Numbers into Movies." *Spatial Vision* 10 (4): 437–42.

Reimers, Stian, Chris Donkin, and Mike E. Le Pelley. 2018. "Perceptions of Randomness in Binary Sequences: Normative, Heuristic, or Both?" *Cognition* 172: 11–25. https://doi.org/https://doi.org/10.1016/j.cognition.2017.11.002.

Sloan, Louise L, William M Rowland, and Adelaide Altman. 1952. "Comparison of Three Types of Test Target for the Measurement of Visual Acuity." *Q Rev Ophthalmol* 8 (1): 4–16.

Vulkan, Nir. 2000. "An Economist's Perspective on Probability Matching." *Journal of Economic Surveys* 14 (1): 101–18. https://doi.org/10.1111/1467-6419.00106.

Wolford, George, Sarah E Newman, Michael B Miller, and Gagan S Wig. 2004. "Searching for Patterns in Random Sequences." *Canadian Journal of Experimental Psychology/Revue Canadienne de Psychologie Expérimentale* 58 (4): 221.

Yellott, John I. 1969. "Probability Learning with Noncontingent Success." *Journal of Mathematical Psychology* 6 (3): 541–75.
